# Application of relative energy deficiency in sport (REDs) guidelines: a scoping review

**DOI:** 10.3389/fspor.2026.1932921

**Published:** 2026-10-19

**Authors:** Paula L. McGahan, Matthew R. Carroll, Prue S. Molyneux

**Affiliations:** 1 Department of Podiatry, School of Allied Health, Auckland University of Technology, Auckland, New Zealand; 2 Active Living and Rehabilitation Research Centre, School of Allied Health, Auckland University of Technology, Auckland, New Zealand

**Keywords:** athlete health, female athlete triad syndrome, healthcare professionals, LEA, RED-S, relative energy deficiency in sport

## Abstract

**Introduction:**

Relative Energy Deficiency in Sport (REDs) is a multifactorial syndrome arising from low energy availability (LEA), leading to physiological, psychological, and performance impairments. Despite established clinical guidelines, integration into research is uneven, demonstrating a research-translation delay. This scoping review aimed to map how REDs guidelines have been operationalised in research and assess the consistency of their application across studies.

**Methods:**

Following the Preferred Reporting Items for Scoping Reviews, a systematic search of Medline, CINAHL, SPORTDiscus, and Scopus was conducted on 21 September 2025.

**Results:**

Thirty-two studies met the inclusion criteria. Twenty-six used mixed Triad/RED-S/REDs terminology, and twenty-six post-2014 studies continued using Triad terms. Five studies followed the Triad framework alone, seventeen studies followed a parallel use of Triad-based models alongside the 2014 and/or later IOC REDs guidelines. LEAF-Q appeared in fifteen studies, only one CAT2. REDs awareness ranged from 10% to 92% across samples.

**Discussion:**

Overall, inconsistency in REDs terminology, screening and operational use of REDs guidelines is apparent in research, highlighting the need for standardised concepts and improved temporal delays in research-translation to support contemporary REDs research and ultimately strengthening athlete health outcomes.

## 1 Introduction

Relative Energy Deficiency in Sport (REDs) is a multifactorial and deleterious syndrome characterised by impaired physiological and psychological functions arising from insufficient energy availability experienced by athletes of all genders (1). Low Energy Availability (LEA), the underpinning mechanism of REDs, is calculated by an imbalance of energy intake minus exercise energy expenditure, occurring when dietary intake is inadequate to meet the demands of exercise and normal physiological processes (2, 3). If left unaddressed, problematic LEA can precipitate REDs, which are associated with a range of impaired physiological functions and performance impairments, including menstrual dysfunction, reduced bone mineral density, compromised musculoskeletal health, such as increased risk of bone stress injuries and impaired tissue repair, and disturbances in cardiovascular health (1, 4).

The concept of REDs was first introduced by the International Olympic Committee (IOC) in 2014 (4), expanding upon the earlier Female Athlete Triad framework. Foundational scientific groundwork in the 1980s by Barbara Drinkwater and colleagues, identified serious health consequences of under fuelling linking to amenorrhoea and bone loss (5). Eventually defined by the American College of Sports Medicine (ACSM) in 1992 (6) and updated in 2007 as the interrelationship between disordered eating, menstrual dysfunction and osteoporosis in physically active females, now known as the Female Athlete Triad (2). Recognising that LEA affects multiple physiological and psychological systems and is not limited to female athletes, the IOC broadened this model in 2014 to establish the REDs framework. The IOC subsequently updated the REDs model in 2018 and again in 2023, further refining its conceptual model, expanding its relevance to male athletes, and providing a more comprehensive clinical assessment and management guidelines (1, 7). Supplementary File 1 outlines the evolution of terminology related to REDs and LEA.

Over the past decade, research into REDs has grown substantially, resulting in a deeper scientific understanding of the condition (8, 9). Nevertheless, awareness and recognition of REDs among athletes and healthcare professionals remain limited, which may leave athletes at risk of potential complications (8). Current consensus highlights the necessity of a proactive approach, with an emphasis on primary prevention, comprehensive knowledge, and early identification of signs and symptoms to optimise athlete health and performance (4, 10–12).

Healthcare professionals and researchers play an important role in bridging the gap between research and practice. Their ability to identify risk factors, educate athletes, and apply effective interventions is critical for safeguarding health and well-being and optimising performance of athletes (4, 10–12). In response to the complexity of REDs, clinical guidelines and evidence-informed frameworks has been developed to assist health practitioners in the identification, management, and prevention of this condition (1, 2, 4, 7, 13–15). These resources promote a proactive, athlete-centred approach and reflect the evolving understanding of REDs and its wide-ranging effects across diverse sporting populations, with an emphasis on early identification. Reviews by Lodge et al. (16) and Burke et al. (17), consistently highlight the importance of the IOC REDs CAT protocol. Recent studies involving youth, elite, winter-sport, and professional team-sport cohorts further suggest that CAT2 may support the early identification and risk stratification of REDs, as well as the assessment of REDs-related health outcomes (18–21).

The 2023 IOC consensus highlights the need for systematic screening and clinical assessment to identify and manage early or mild cases of LEA and REDs to protect athlete health (1). The Low Energy Availability in Females Questionnaire (LEAF-Q) and Low Energy Availability in Males Questionnaire (LEAM-Q) are self-report tools designed to identify athletes at risk of LEA, capturing key physiological and performance associated symptoms (12). These questionnaires form the initial step of the IOC REDs Clinical Assessment Tool, Version 2 (CAT2) (22). The CAT2 provides a structured, clinical guided framework for risk stratifications and participation decision making using a traffic light system. Together, these tools facilitate early detection and evidence-based intervention, aligning with the IOC 2023 guidelines for research and clinical practice.

Despite the availability of REDs-related conceptual frameworks, screening tools, and IOC guidance, it remains unclear how consistently these resources have been incorporated into published research. Emerging evidence suggests that awareness and knowledge of REDs among health practitioners may be limited, which could hinder early recognition and appropriate intervention (8). This may reflect a broader knowledge-translation gap, whereby established evidence and clinical resources are not yet being consistently integrated into research design, measurement, or interpretation.

Although individual studies have applied IOC REDs guidance in athlete populations and examined REDs-related outcomes, no review has synthesised how REDs-related frameworks, tools, and concepts have been operationalised across the research literature. It is unclear how consistently studies define REDs-related terminology, apply screening or assessment approaches, and incorporate multidisciplinary perspectives. This may represent a gap in temporal delays in research translation rather than evidence of inconsistent clinical implementation.

To address this gap, this scoping review aimed to map how REDs guidelines have been operationalised in research and assess the consistency of their reference across studies. Specifically, the review examined the use of REDs-related terminology, guidelines and tools, and awareness representation among athletes, health practitioners and athlete entourage. By mapping these characteristics, the review identified inconsistencies and highlights opportunities for future research to achieve stronger methodological alignment with contemporary IOC guidance.

## 2 Methods

### 2.1 Positionality statement

For this scoping review, terminology was aligned with the most recent IOC 2023 consensus statement on REDs. However, given the evolution of the concept from the Female Athlete Triad to REDs, the original terminology used within included studies was retained when describing source literature to ensure accurate representation of the evidence base. As the purpose of this review was to map the breadth, range, and nature of published research relating to REDs, low energy availability (LEA), and the Female Athlete Triad, rather than to evaluate clinical implementation, the term “guideline/framework application” was defined as the use of REDs- or Triad-related frameworks, screening instruments, or assessment tools within the research literature. This included their explicit use in study design, participant classification, measurement, data analysis, or interpretation of findings. Examples included the IOC REDs guideline, Female Athlete Triad model, Low Energy Availability in Females Questionnaire (LEAF-Q), Disordered Eating Assessment for Athletes Questionnaire (DEAQ), and the IOC REDs Clinical Assessment Tool (CAT/CAT2). Importantly, this definition refers to application within research settings and should not be interpreted as evidence of real-world clinical implementation, adherence to guideline recommendations, or use within routine healthcare practice.

The framework proposed by Arksey and O'Malley (23) was used to guide the scoping review methodology. The method involved five stages: identifying the research question, identifying relevant studies, selecting studies, charting the data, and collating, summarising, and reporting the results (23). To ensure methodological rigour and transparency, this review was conducted and reported in accordance with the Preferred Reporting Items for Systematic Reviews and Meta-Analyses extension for scoping reviews (PRISMA-ScR) checklist (24–26). The PRISMA-ScR checklist is displayed in Supplementary File 2. Consistent with scoping review methodology, no critical appraisal of included studies was undertaken, as the purpose of this review was to map the literature rather than evaluate study quality, intervention effectiveness, or real-world implementation of REDs-related guidance. Furthermore, no protocol was prospectively registered for this scoping review.

### 2.2 Search strategy

Studies were included if they were original research articles that referred to REDs, energy availability, LEA, TRIAD or male athlete triad; discussed a sporting code, athletic population, addressed a risk factor to REDs; referenced an IOC guideline or framework relating to TRIAD or REDs; and/or involved a healthcare provider for REDs management. The scoping review searched major academic databases using a structured combination of subject headings and keywords related to REDs. A 10-line Boolean search strategy using controlled vocabulary (e.g., MeSH terms) and free-text keywords and was adapted for use across multiple databases. Search terms included the controlled vocabulary term “Relative Energy Deficiency in Sport” as well as keyword variants, including “Relative energy deficiency in sport” OR “REDs” OR “RED-S” OR “Low energy availability” OR “LEA”. To capture studies involving healthcare practitioners, and interdisciplinary care, additional terms included (Interdisciplin* OR Multidisciplin* OR “health professional” OR “health practitioner” OR “health specialist” OR Podiatr* OR Dietician OR Nutritionist OR “Sport* Physician” OR Endocrinologist OR Physio*. Terms relating to guideline application and clinical processes were also included, such as Guidelin* OR Recommen* OR Asses* OR Tool* OR Screen* OR Treat* OR Prevent*. Articles were identified through a comprehensive search of titles and abstracts across key electronic databases. Searches were conducted in September 2025. The databases MEDLINE, CINAHL, SPORTDiscus, and Scopus were systematically searched from database inception to September 2025. The search strategy and broad-ranging search terms were developed collaboratively by two authors (PLM and PSM) and a Senior Research Librarian at Auckland University of Technology, ensuring reproducibility and adherence to best-practice recommendations for scoping reviews. The complete search strategy is provided in Supplementary File 3.

The selection process comprised two stages. First, two authors (PLM and PSM) independently screened the titles and abstracts. We then retrieved full texts of potentially eligible articles and assessed them against the inclusion criteria. Articles were excluded if they were not published in English, were opinion pieces, commentary letters, review articles, or non-human studies. Case reports and case series were also excluded due to the risk of selection bias. The two reviewers (PLM and PSM) discussed any eligibility disagreements until consensus was reached. If consensus could not be achieved, a third reviewer (MC) was available for consultation, although this was not required.

All titles and abstracts identified from the search were imported into EndNote 21 (Thomson Reuters, Philadelphia, PA USA) for reference management and duplicate detection. Screening procedures, including duplicate removal, title and abstract screening, and full-text review, were conducted using Rayyan systematic review software (Rayyan; Qatar Computing Research Institute) (27). The reference lists of all included articles were subsequently hand-searched to identify additional relevant studies. Any potentially eligible studies identified through this process were retrieved and screened against the predefined inclusion criteria using Rayyan. A PRISMA-ScR flow diagram (Figure 1) summarises the study-selection process, including database yields, automated filtering, deduplication using EndNote and Rayyan, title and abstract screening, full-text assessment, and reasons for exclusion. The final diagram reflects all steps undertaken in accordance with PRISMA-ScR guidance and reports the exact number of records screened, excluded, and included.

**Figure 1:**
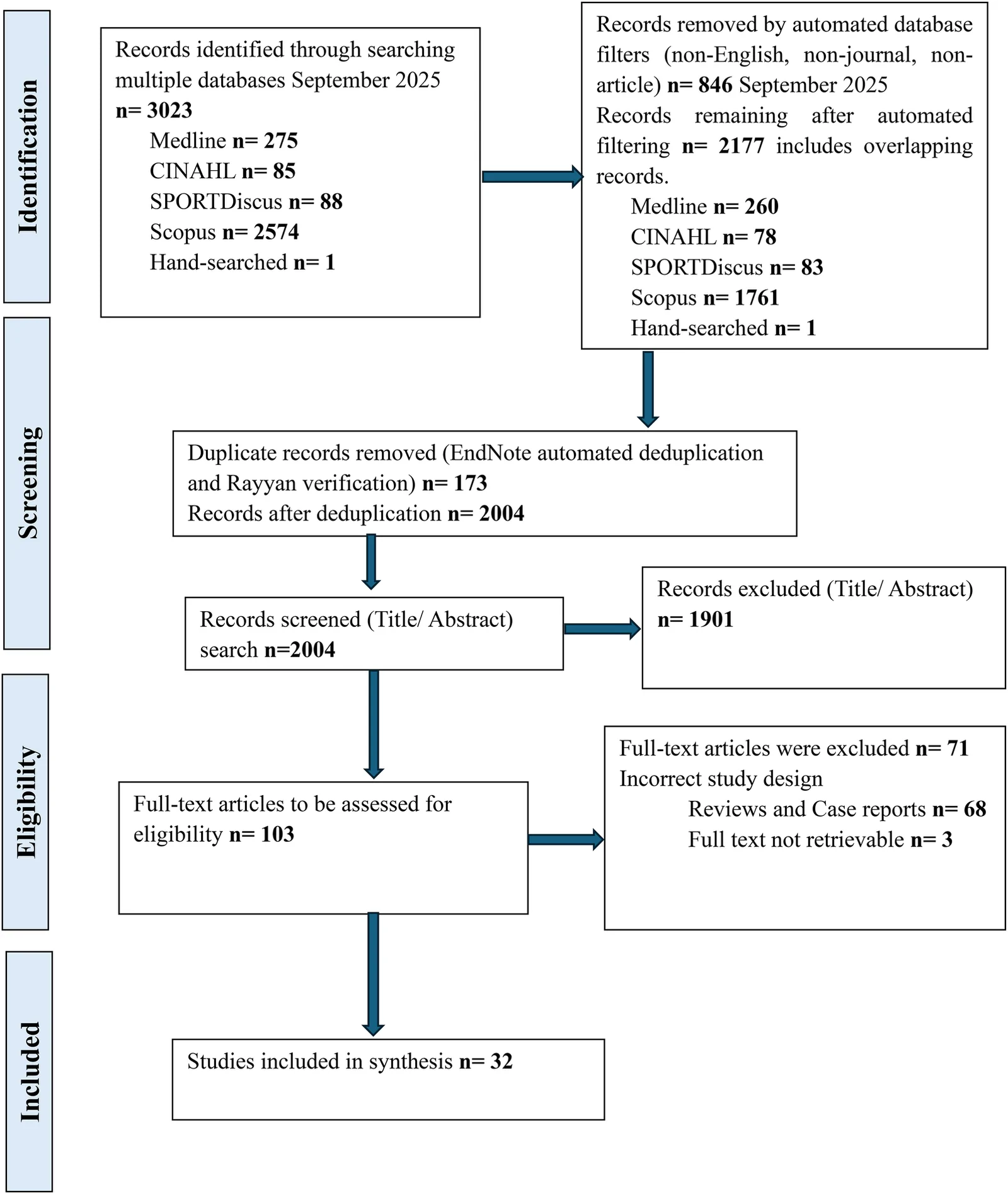
Scoping review PRISMA flow diagram selection of sources of evidence.

### 2.3 Data extraction

Three reviewers (PLM, PSM, MC) developed a structured data-charting form collaboratively. The first author (PLM) independently extracted all data from the eligible articles for data synthesis. For each study, the following data were extracted: study aim, country of research, study design, participant demographics, including sample size, gender distribution, and whether participants were athletes, health professionals, or both. Additionally, for studies involving health professionals, the terminology used (LEA, Triad, REDs), guideline or protocol utilisation, multidisciplinary roles, and reported competency or awareness levels were reported. For studies involving athlete populations, we reported health care provider involvement, use of guidelines or prevention tools, details of guideline application, and referral practices. The structured data-charting form for studies involving health professionals and athlete-focused is presented in Supplementary File 4. Consistent with scoping-review guidance Mak and Thomas (28), the draft charting forms were collaboratively developed and pilot-tested to ensure clarity, consistency, and completeness before full data extraction commenced. Missing data were recorded as “not reported/N/R.” Consistent with JBI and PRISMA-ScR guidance, no thematic analysis or meta-analysis was conducted. Instead, data were summarised using, descriptive counts and percentages, and tables mapping guideline use and operationalisation (29).

## 3 Results

### 3.1 Selection of studies

A total search yielding 3,023 records were identified for screening. Following title and abstract screening and full-text review, 32 studies met the inclusion criteria and were included in the final synthesis (Figure 1).

### 3.2 Characteristics of included studies

The 32 included studies combined a total sample size of 5,513 (928 males, 4,164 females, 421 did not specify), including 1,785 professionals and 3,728 athletes. Publication dates ranged from 2008 to 2025. Table 1 outlines study characteristics, participant demographic characteristics and all key interests of all included studies.

**Table 1:** Participant demographics, study characteristics and key interests.

Study (citation)	Number (Female: Male: N/R)	Sporting code	Professional type	Awareness	Terminology	Guideline/frameworks	Validated screening tool	Key interests
Athlete samples								
Amoruso et al. (41)	288 (288:0)	National Italian Female Athletes	N/R	N/R	Refers to TRIAD	TRIAD	N/R	­ Exclusion of REDs Terminology and Framework ­ Despite being published in 2024 ­ Implemented non-validated screening tool ­ Acknowledges Translation and Consensus Lag ­ - Reflects frameworks are yet to be operationalised across contemporary research.
Pereira de Maria et al. (44)	147 (147:0)	Mixed Endurance	N/R	N/R	Refers to TRIAD	TRIAD	LEAF-Q	­ Study is Operationalised around the Female Athlete Triad framework ­ - Acknowledge the lack of validated screening tools may explain LEAF-Q rather than newer CAT
Drew et al. (37)	81 (55:26)	Australian Olympic Athletes	N/R	N/R	Refers to both TRIAD & RED-S	TRIAD & IOC 2014	LEAF-Q	- Discusses both terms in parallel TRIAD & RED-S - Despite introduction of the RED-S framework in 2014, researchers continued to overlap with Triad model. Highlights a natural lag in published research - Researchers may align with the broader IOC frameworks yet, includes both males and females, and continued with TRIAD
Dimitriou et al. (39)	21 (21:0)	Elite Lightweight Rowers	N/R	N/R	Refers to TRIAD	TRIAD	N/R	- Highlights that early 2014 research was grounded strictly in the Triad paradigm - Discusses and compares to Male Athletes
Drew et al. (38)	132 (85:47)	Australian Olympic Athletes	N/R	N/R	Refers to LEA	N/R	LEAF-Q	- Despite introduction to RED-S, study focuses strictly on the isolated concept of LEA and TRIAD - Highlights a transitional phase in research where screening for LEA was well-established and newer terminology and guidelines of RED-S had not yet been operationalised
Edama et al. (48)	116 (116:0)	Collegiate Female Athletes	N/R	N/R	Refers to both TRIAD & RED-S	TRIAD	N/R	- While they discuss the newer RED-S frameworks (IOC 2014/2018), the authors explicitly noted that “RED-S is insufficiently supported by scientific research to warrant adoption at this time” - Felt TRIAD had stronger evidence-based clinical guidelines - Though conclude their study by noting RED-S - Authors rely on Triad-specific tools for their methodology; they still use RED-S terminology
Gillbanks et al. (36)	12 (8:4)	Rowers	N/R	N/R	Refers to both TRIAD & RED-S	IOC 2014	N/R	- Despite available validated tools, none were used LEAF-Q, or CAT. - Explain that while the traditional Triad model focused on females the newer RED-S terminology is broader
Hajian and Mohaghegh (51)	25 (25:0)	Dancers	N/R	N/R	Refers to both TRIAD & RED-S	N/R	N/R	­ While the study conceptually references TRIAD and the IOC 2018 Consensus Statement, it does not operationalise these frameworks to screen, or diagnose ­ The study does not reference the updated IOC 2023 guidelines or the revised “REDs” terminology ­ Uses RED-S interchangeably with TRIAD ­ Methodological choices often result in studies that rely on older paradigms
Jesus et al. (35)	207 (83:124)	Elite Runners	N/R	N/R	Refers to both TRIAD & RED-S	TRIAD & IOC 2014/18	LEAF-Q	­ Study demonstrates a parallel approach, grounding study in both TRIAD and IOC
Logue et al. (34)	833 (833:0)	Active Athletes	N/R	N/R	Refers to both TRIAD & RED-S	TRIAD & IOC 2014/18	LEAF-Q	­ The study demonstrates a highly overlapping, mixed approach to terminology and frameworks. ­ The authors design and run their screening using the Triad framework, however, the authors discuss and frame their findings in the context of the IOC ­ Despite the IOC publishing the CAT in 2015 authors did not use, yet available
Łuszczki et al. (49)	34 (34:0)	Youth Female Football Players	N/R	N/R	Refers to both TRIAD & RED-S	TRIAD & IOC 2014	LEAF-Q	­ This study highlights a significant gap in their consistent application, making early screening frameworks essential to prevent future, long-term clinical issues
Melin et al. (47)	40 (40:0)	Female Endurance Athletes	N/R	N/R	Refers to both TRIAD & RED-S	TRIAD	LEAF-Q	­ Operationalised the Triad framework for diagnostic groupings but integrated the newly published IOC 2014 RED-S framework into discussion
Melin et al. (13)	45 (45:0)	Female Endurance Athletes and Dancers	N/R	N/R	Refers to TRIAD	TRIAD	LEAF-Q	- Was designed and written prior to the release of the first IOC consensus statement, relying on TRIAD
Melin et al. (46)	25 (25:0)	Female Endurance Athletes	N/R	N/R	Refers to both TRIAD & RED-S	TRIAD & IOC 2014	LEAF-Q	­ Integrated both Triad and RED-S (IOC 2014) in parallel from its introduction through its discussion
Mora-Fernandez et al. (33)	46 (24:22)	Spanish Climbers	N/R	N/R	Refers to both TRIAD & RED-S	TRIAD & IOC 2014	N/R	­ Conceptually frame and transition Triad, “Male Athlete Triad”, and RED-S models ­ Cite publications using the newer “REDs” acronym in their bibliography ­ Although this 2024 study extensively references the Athlete Triad and RED-S conceptual models as well as the IOC consensus guidelines
Nicholas and Grafenauer (32)	63 (58:5)	Pre-professional Dancers	N/R	Awareness among dancers: 73.4% were aware of LEA, 56.3% were aware of Triad, and 53.1% were aware of REDs, and 18.8% perceived it as normal for female dancers to not have periods	Refers to mixed REDs RED-S and TRIAD	TRIAD & IOC 2023	LEAF-Q & CAT	­ Uses updated IOC 2023 REDs guidelines and screening parameters ­ Parallel discussion of both REDs and Triad frameworks
Paduan Joaquim et al. (31)	17 (8:9)	Elite Paralympic Sprinters	N/R	N/R	Refers to mixed TRIAD, RED-S & REDs	TRIAD & IOC 2014	N/R	­ Interchangeably uses IOC and TRIAD, both syndromes side-by-side
Tektunalı Akman et al. (50)	83 (83:0)	Young Elite Female Endurance Athletes	N/R	Among the athletes, their knowledge of sports nutrition was low, scoring less than 50% out of 83points on the Sports Nutrition Knowledge Questionnaire (SNKQ).	Refers to mixed TRIAD, RED-S & REDs	TRIAD	LEAF-Q	­ This 2024 study highlights a common lag in the integration of current REDs frameworks ­ Despite being published well after 2014, the study does not operationalise updated IOC guidelines.
Verhoef et al. (30)	12 (12:0)	Endurance athletes	N/R	N/R	Refers to both TRIAD and RED-S	IOC 2014/2018	N/R	­ The study discusses the evolution from the Female Athlete Triad to the IOC 2014 and 2018 RED-S consensus frameworks ­ It does not operationalise a clinical framework to assess the participants ­ The paper mentions the RED-S CAT (Clinical Assessment Tool), but it was not applied
de Borja et al. (40)	1,000 (1,000:0)	Female Athletes	N/R	N/R	Refers to both TRIAD and RED-S	TRIAD & IOC 2014/18	N/R	­ The study grounds its context in both Triad and the IOC RED-S 2014 & 2018. ­ RED-S and Triad interchangeably
Meyer et al. (45)	58 (56:2)	Endurance Athletes	N/R	N/R	Refers to both TRIAD & REDs	IOC 204, 2018 & 2023	CAT	­ They could not report active CAT2 scores because CAT2 was not active during the 2019–2022 clinical data assessment window. ­ Highlights the challenge of incorporating newer guidelines into research ­ The lag between academic publishing and clinical documentation meant that missing parameters ­ Parallel use of IOC 2014, IOC 2018, and IOC 2023 guideline
Courtney et al. (42)	122 (122:0)	Amateur Female Gaelic Athletes	N/R	Among Gaelic games athletes, 59.8% were aware of dietary and exercises impacts on LEA. 18.0% were aware of LEA risk in sport.	Refers to mixed TRIAD, RED-S & REDs	TRIAD & IOC 2014, 2018 & 2023	LEAF-Q	­ Major gaps in how established guidelines and tools are operationalized ­ By applying this mixed-framework standard, the researchers demonstrate a stark gap between international guidelines
Heikura et al. (18)	213 (143:70)	Elite Athletes	N/R	Awareness was significantly higher among athletes categorized into higher REDs risk severity groups (red/orange light)	Refers to mixed RED-S & REDs	IOC 2023	LEAF-Q & CAT2	­ This study represents the first empirical validation and field implementation of the 2023 IOC REDs CAT2 framework in a large elite cohort ­ It illustrates how IOC guidelines are translated into practice while highlighting real-world implementation challenges
Professional samples								
Bowler et al. (52)	55 (50:5)	N/R	Australian Sports Dietitians	Among sports dieticians, mean confidence in identifying LEA was 7.4/10 ± 1.3 on a10-pointscale	Refers to both TRIAD and RED-S	TRIAD & IOC 2018	LEAF-Q	­ They explicitly link it as the underlying contributor to both the Triad and RED-S ­ developed using the Australian Institute of Sport (AIS) RED-S Athlete Management Pathway Infographic ­ Notable gap between conceptual frameworks and real-world implementation—IOC and EA
Kroshus et al. (53)	285 (102:125) (58 NR)	N/R	Head Athletic Trainers	Among athletic trainers, 98% were aware of the Triad, and 33% were aware of REDs	Refers to both TRIAD and RED-S	TRIAD & IOC 2014	N/R	­ The paper operationalises the definitions of the TRIAD and IOC RED-S ­ Although the IOC 2014 had recently introduced the expanded RED-S framework, 32.98% of collegiate head athletic trainers (ATs) had heard of RED-S. In contrast, 98.61% were familiar with the older “Female Athlete Triad” terminology ­ The authors point out that newer conceptualisations take a long time to trickle down into translation of education ­ Acts as a historical baseline showing the immediate challenges of disseminating the IOC 2014 consensus
Pantano (54)	205 (103:102)	N/R	Physical Therapists	TRIAD: 61% of respondents *perceived* themselves as having adequate knowledge. 21% could accurately list all three components of the Triad	TRIAD	TRIAD	N/R	­ Historical baseline representing the pre-REDs era ­ Even though the Triad had received significant exposure in scientific journals by 2008, only 21% of surveyed physical therapists could accurately identify all three components of the Triad ­ This gap occurred despite 61% of those therapists *perceived* that they had adequate knowledge
Tenforde et al. (55)	163 (88:74)	N/R	Sports Medicine Professionals: Physician, Allied health, Nurse., Physical Therapist, Athletic Trainer)	Awareness among sport medicine professionals: 76% were aware of Triad, 29% were aware of REDs. 33% felt comfortable treating Triad and 13% felt comfortable treating REDs.	Refers to both TRIAD and RED-S	TRIAD & IOC 2014, & 2018	N/R	­ The study operationalised the Triad framework alongside the RED-S framework 2014 IOC and the 2018 IOC ­ While this study contextualizes its research within the evolving conceptual frameworks of the Triad and RED-S, it highlights a substantial gap in translating these concepts ­ Despite the publication of international guidelines, clinical comfort with and awareness of the RED-S framework remains low
Verhoef et al. (56)	799 (496:303)	N/R	Physiotherapist, Physicians, Surgeons, Gynecologist	Among healthcare providers, 22% were aware of REDs. Awareness among professional groups: sports physicians 92%, orthopedic surgeons 23%, physiotherapists 18%, gynecologists 11%, and general practitioners 10%. Among healthcare providers, 57.6% felt competent discussing REDs, and 7.4% felt qualified to treat REDs.	Refers to both TRIAD and RED-S	Triad & IOC 2014 & 2018	N/R	­ Operationalised the concepts introduced in the IOC 2014 ­ Though there is a severe lag between the publication of international consensus frameworks and their integration ­ Sports physicians have integrated the IOC 2014/2018 frameworks (92.9% awareness), the primary care professionals who act as first-line contacts for athletes remain unaware of RED-S general practitioners (10.1%) and physiotherapists (18.5%) ­ This study underscores that having conceptual frameworks is not enough; active integration into education is critically required to close the gap between research and clinical care
Warrick et al. (11)	161 (Gender N/R)	N/R	Physicians	Among physicians; 48% correctly identified Triad and 32% identified REDs features; orthopedic surgeons reported 100% awareness of both conditions.	Refers to both TRIAD and RED-S	Triad & IOC 2014 & 2018	N/R	- The study's findings directly demonstrate significant inconsistency and gaps in how well these clinical resources and frameworks are integrated - Only 48% of the physicians surveyed could correctly identify the three Triad criteria and only 32% could correctly identify the features of RED-S - This highlights that despite consensus frameworks existing since 2014 and 2018, their dissemination and consistent incorporation into non-sports-specialty medicine remain highly fragmented
Mixed athlete and professional samples								
Pai et al. (15)	174 (N/R)	(*n* = 77) Active Individuals	(*n* = 97) Dietitians, Nutritionists, Physiotherapists, Physicians and Personal trainers	Awareness among health professionals: 41.4% had heard of Triad, 20.1% had heard of LEA, and 15.5% had heard of REDs.	Refers to both TRIAD and RED-S	Triad & IOC 2014 & 2018	LEAF-Q	­ Highlighted inconsistencies across research designs between established guidelines and research tools ­ To address this inconsistency, formulated a new validated questionnaire to provide research with a consistent evidence base questionnaire aligned with IOC
O’Donnell et al. (8)	21 (N/R)	(*n* = 13) Netball Players	(*n* = 8) (4 Coach, 2 Doctor, 2 Physiotherapist)	Among players, only one athlete (7.7%) had heard of REDs; Among the coaches 0% reported awareness of the REDs. Among the medical professionals 75% had heard of REDs and Triad.	Refers to both TRIAD and RED-S	Triad & IOC 2014	N/R	­ The study asked medical personnel about their awareness of the RED-S CAT (Clinical Assessment Tool); however, none of the medical professionals had ever seen or used the tool prior to the study ­ gap between the existence of official guidelines and their actual translation
Gillbanks et al. (43)	24 (14:10)	(*n* = 12 (6:6)) Rowers	(*n* = 12 (8:4)) Physiotherapists	Among rowers 25% had heard of REDs, 50% had no knowledge of REDs. Among physiotherapists, 42% knew REDs and 58% had no knowledge of REDs. 67% of physiotherapists believed their lack of REDs knowledge may result in health implications.	Refers to RED-S	Triad & IOC 2014 & 2018	N/R	­ However, in the discussion, the authors explicitly reference and advocate for the clinical use of the CAT Assessment Model ­ Despite the long-standing availability of the IOC 2014 and 2018 frameworks and clinical tools, they have not consistently reached practitioners or athletes ­ Over half of the physiotherapists (58%) had never even heard of RED-S
Total:	5,513 (4,164:928:421)	3,728 Athletes	1,785 Professionals					

Citations presented in this table correspond directly to the references cited in the main manuscript.

*n*, Number of participants; M, Male; F, Female; T, Total; N/R, not reported; RED-S, relative energy deficiency in sport; REDs, relative energy deficiency in sport; Triad, the female athlete triad; LEA, low energy availability; IOC, International Olympic Committee; CAT, IOC clinical assessment tool; CAT2, IOC clinical assessment tool version 2; LEAF-Q, low energy availability in females questionnaire.

### 3.3 Participant populations

Twenty-six studies included athlete participants at risk of developing REDs (8, 13, 15, 18, 30–51). Nine studies involved members of the athlete healthcare team, focusing on how they identify LEA and manage REDs (8, 11, 15, 43, 52–56). Three of these (8, 15, 43) included both athletes and health professionals. Studies involving health professionals and athletes are presented in Supplementary File 4. The three studies that include both athletes and health professionals are included in both files.

#### 3.3.1 Athlete and sporting codes

Of the thirty-two included studies, twenty-six studies involved athletic populations across a wide range of sporting disciplines. Nine studies involved mixed sport cohorts comprising athletes from multiple disciplines (e.g., basketball, swimming, running, dance, rowing) without a dominant sporting code (13, 18, 34, 37, 38, 41, 44, 45, 48). Seven studies focused specifically on endurance sports such as rowing, cycling, and running (30, 35, 36, 39, 43, 46, 47). Four studies examined team sport athletes, including Gaelic football, camogie, football, and netball (8, 42, 49, 50). Two studies investigated athletes from aesthetic sports, specifically dance (32, 51). One study included Paralympic track and field athletes (31) and one examined rock-climbers (33). Two additional studies included general athlete populations that were not further defined (15, 40).

#### 3.3.2 Professional disciplines

Nine of the thirty-two included studies examined the involvement of professional disciplines in the management of REDs (8, 11, 15, 43, 52–56). Four studies focused on a single professional group, including sports dietitians, physicians, physical therapists, or athletic trainers (11, 52–54). Two studies explored multidisciplinary team approaches, involving combinations of physicians, allied health practitioners, nurses, physical therapists/physiotherapists, surgeons, gynaecologists, and athletic trainers (55, 56). An additional three studies included mixed populations comprising health professionals, athlete entourage members, and athletes. Of the studies that incorporated both athletes and professional groups, one examined physiotherapists alongside rowers (43), one included netball players together with a coach, doctor, and physiotherapist (8), and another involved a multiprofessional cohort: dietitians, nutritionists, physiotherapists, physicians, and personal trainers, working with a general athletic population (15).

### 3.4 Evolution of terminology

Across the 32 included studies, terminology related to LEA and its associated conditions varied considerably (Table 1). All studies explicitly identified LEA as the underlying aetiology, despite using differing or overlapping diagnostic terms. Twenty-nine studies (8, 11, 15, 18, 30–38, 40–53, 55, 56) were published after the introduction of RED-S in 2014. Of these, twenty-six studies (8, 11, 15, 30–37, 40–42, 44–53, 55, 56) continued to use Triad-based terminology. Nine studies (18, 32, 33, 41, 42, 45, 50, 51, 56) were published after the introduction of the 2023 IOC update of the REDs framework; however, only five of the studies (18, 32, 42, 45, 50) adopted the updated hyphen-free “REDs” terminology aligned with the most recent guidelines (1). One study (48) notably diverged from the progression to REDs, explicitly rejecting the conceptual shift toward REDs and retaining exclusive use of the term “Triad”. Most studies (*n* = 25) (8, 11, 15, 18, 30–37, 40, 42, 45–53, 55, 56) used mixed or parallel terminology, combining “Female Athlete Triad,” and/or “RED-S,” and/or “REDs.” Table 1 summarises the terminology used across all included studies.

### 3.5 Operationalisation of clinical guidelines

Relative Energy Deficiency in Sport was introduced in the 2014 IOC consensus statement. Three studies (13, 39, 54) were published prior to the 2014 update and, accordingly, all followed the earlier Female Athlete Triad guidelines. Twenty-nine studies (8, 11, 15, 18, 30–38, 40–53, 55, 56) were published after the 2014 IOC RED-S guidelines were released (4). Among these, five studies (41, 44, 47, 48, 50) followed the Triad framework alone, seventeen studies (8, 11, 15, 31–35, 37, 40, 42, 43, 46, 49, 52, 53, 55) followed a parallel use of Triad-based models alongside the 2014 and/or later IOC REDs guidelines, and five studies (18, 30, 36, 45, 56) followed the 2014 and/or later IOC RED-S/REDs guidelines alone. A further two studies (38, 51), while conceptually mentioned frameworks, they did not operationalise any frameworks. The most recent IOC REDs update was released in 2023 (1). Among the 32 included studies, nine studies (18, 32, 33, 41, 42, 45, 50, 51, 56) were also published in or after 2023, though only four studies (18, 32, 42, 45) followed the 2023 REDs guidelines. Table 1 summarises the application of clinical guidelines across all included studies.

### 3.6 Screening and clinical assessment tools

Among the 32 studies, the Low Energy Availability in Females Questionnaire (LEAF-Q) and the Clinical Assessment Tool (CAT) were the most referred to validated screening and assessment tools (Table 1). Fifteen studies (13, 15, 18, 32, 34, 35, 37, 38, 42, 44, 46, 47, 49, 50, 52) referred to the LEAF-Q and two studies (32, 45) referred to the CAT. And only one study (18) referred to the updated Clinical assessment tool version 2, CAT2, despite introduced in 2023.

### 3.7 Methodology to assess knowledge and awareness of Triad and REDs

Thirteen studies (8, 11, 15, 18, 32, 42, 43, 50, 52–56) surveyed knowledge, awareness, or competency related to the Triad and REDs among healthcare professionals, athletes, and members of the athlete entourage. Of these, ten studies (11, 18, 32, 42, 50, 52–56) used quantitative methodologies, primarily online questionnaires or electronic surveys, to assess knowledge, awareness, and/or confidence in recognising LEA, the Triad, or REDs. The remaining three studies (8, 15, 43) used qualitative semi-structured interviews to explore perceptions, experiences, competency, and knowledge related to the Triad, LEA, and REDs among both athlete and professional groups. Two studies (11, 50) implemented a longitudinal survey design, conducting initial and follow-up assessments to measure changes in knowledge, awareness, and competency after an educational intervention.

### 3.8 Perceived levels of competency among health practitioners and athlete entourage

Thirteen studies (8, 11, 15, 18, 32, 42, 43, 50, 52–56) surveyed knowledge, awareness, or competency related to the Triad and REDs among healthcare professionals, athletes, and members of the athlete entourage. Among the thirteen studies, seven studies (8, 11, 15, 32, 42, 53, 55) compared Triad and REDs knowledge. Three studies (18, 43, 56) surveyed REDs knowledge alone, and one study (54), prior to the introduction of REDs surveyed Triad alone.

Four studies (11, 15, 55, 56) reported substantial variability in knowledge across professional groups. In a large multi-professional survey (56), only 22% of 799 providers were aware of REDs, with the highest awareness among sports medicine physicians (92%). Awareness was considerably lower among orthopaedic surgeons (23%), physiotherapists (18%), gynaecologists (11%), and general practitioners (10%). The same study found limited confidence among physicians and physiotherapists in recognising and managing REDs: while 57.6% felt competent discussing potential underlying issues, only 7.4% felt qualified to provide treatment. Similarly, another study (55) reported that most health professionals (76%) were more aware of the Triad than REDs (29%), and that participants felt more comfortable managing athletes with the Triad (33%) than with REDs (13%). A third study (11) found that 48% of physicians correctly identified the components of the Triad, whereas 32% identified key characteristics of REDs; notably, orthopaedic surgeons reported 100% awareness of both conditions. In a New Zealand cohort (15) familiarity was reported as variable (Triad: 41.4%; LEA: 20.1%; REDs: 15.5%).

Three additional studies (43, 52, 53) showed mixed competency among other practitioner groups. Athletic trainers demonstrated high awareness of the Triad (98%) but much lower awareness of REDs (32%) (53). Sports dietitians reported strong confidence in identifying LEA (mean 7.4/10), and 55% indicated that LEA assessment formed part of routine practice (52). Conversely, physiotherapists demonstrated comparatively low knowledge and confidence related to REDs (43).

Four studies (8, 32, 42, 50) identified low awareness of REDs among athletes and entourage. In one study of a professional netball club (8) only one of thirteen athletes had heard of REDs, and coaches reported no awareness. Gaelic games athletes (42) showed moderate awareness of LEA-related health impacts (59.8%). Pre-professional dancers (32) demonstrated higher levels of awareness (LEA: 73.4%; Triad: 56.3%; REDs: 53.1%), whereas young endurance athletes (50) exhibited low baseline sports nutrition knowledge prior to receiving education. One study (18) compared awareness levels between males and females and also examined awareness across REDs risk-severity categories. Table 1 summarises LEA, Triad, and REDs awareness across studies.

### 3.9 Professional roles and referral pathways in athlete care

Nine of the 32 studies (8, 11, 15, 43, 52–56) explored the roles and responsibilities of various healthcare professionals and members of the athlete entourage within multidisciplinary teams for detecting, managing, and preventing REDs or LEA. Seven studies (8, 11, 43, 52, 53, 55, 56) identified recognition of REDs as a primary responsibility across these groups. One study (56) reported that physicians tended to adopt a “wait-and-see” approach when managing female athletes presenting with menstrual disturbances. Another study (53) highlighted that incorporating screening into pre-participation examinations was considered a key role. Across the seven studies identifying practitioner roles, five studies (8, 43, 52, 53, 56) emphasised responsibility for referring athletes to other professionals. Two studies (11, 56) also reported that initiating oral contraceptives was sometimes considered appropriate when menstrual dysfunction was recognised.

Among the nine studies, five studies (8, 43, 52, 53, 56) described established referral pathways. All five reported referral to dietitians. One study (52) noted that dietitians frequently received referrals from sports physicians for LEA management. Two studies (43, 56) investigated physiotherapists; one study (43) found physiotherapists felt insufficiently skilled to manage REDs and therefore “signposted” athletes to GPs, dietitians, or psychologists; the other (56) reported physiotherapists contacted the athlete's GP directly or advised the athlete to do so. The same study (56) also involved orthopaedic surgeons and sports physicians, reported common referrals to dietitians, sports physicians and gynaecologists. A further study noted that athletic trainers primarily referred athletes to sports medicine physicians and sports dietitians (53). One study (8) involving mixed professionals and members of the athlete entourage (physiotherapists, doctors, coaches) did not identify a specific referral pathway but emphasised the need for multidisciplinary team involvement.

## 4 Discussion

This review synthesised findings from 32 studies examining athletes' and practitioners' experiences, screening practices, terminology, and the representation of REDs-related guidance in literature. Across these studies, inconsistency was evident in the terminology used, screening approaches, and how REDs-related frameworks were followed. Variation was also observed in knowledge and awareness of the Triad and REDs among athletes, health practitioners, and members of the athlete entourage. Collectively, these findings indicate that advances in REDs evolution have not yet been consistently adopted in research.

Although the term RED-S was introduced in 2014, and updated to REDs in 2023, most studies continued to use Triad-related terminology, evident with over two-thirds (*n* = 25) of studies using overlapping, mixed, or outdated terminology. This narrow conceptualisation may reflect a historical bias toward female and elite athlete samples, and therefore limiting recognition of risk in male, para-athlete, and recreational populations when framing research questions. Gallant, et al. (57) similarly reported substantial variability in terminology noting that inconsistent definitions hinder standardised guideline application and may lead to underestimation of REDs prevalence. The presence of overlapping, mixed, or outdated terminology can be a typical indicator of a conceptual transition phase in academic literature (58). Together, signalling ongoing conceptual confusion, and natural lag in published research, potentially limiting recognition of the multisystem nature of REDs (1).

Most included studies not only referred predominantly to Triad terminology but also anchored their research context in Triad guidelines, either solely or through a parallel approach of both Triad and IOC guidelines. Despite 90% of the studies (*n* = 29) included in this review being published after the introduction of IOC REDs guidelines, and most having availability to the updated recommendations, IOC REDs was not consistently followed. This review identified gaps in how established guidelines are represented in the literature. More than a decade after REDs was introduced, many studies continue to apply mixed guidelines, highlighting a persistent disconnect between consensus recommendations and research design. While prior literature acknowledges that newer conceptual models can take time to translate into published research (53), the extent and duration of this historical lag suggest a deeper conceptual and methodological delay within the scientific community. In our review, only one study by Heikura et al. (18) formally followed the IOC 2023 guidelines. However, even this study required minor methodological adaptations because data collection occurred from 2021 to 2023. While such variation across studies may contribute to conceptual uncertainty in the published literature, it does not necessarily demonstrate practitioner confusion or clinical misapplication.

Consistent with the predominance of Triad terminology and Triad-based guidelines among the included studies, awareness patterns reported among healthcare practitioners reflected a similar trend. Across the studies that surveyed healthcare practitioners, awareness of the Female Athlete Triad was consistently higher than awareness of REDs, with awareness of REDs remaining comparatively low. The highlighted challenges posed by longer existing historical guidelines and demonstration of contemporary REDs guidelines are yet to be consistently operationalised within research. For example, in a 2022 survey of 174 health practitioners, 41% had heard of the Triad compared with only 15% who had heard of REDs (15). In 2023, among 161 practitioners surveyed, 48% were aware of the Triad and 32% were aware of REDs (11). By 2024, in a larger sample of 799 practitioners, only 22% reported awareness of REDs (56). Comparatively, a 2025 review had reported similar findings of awareness of REDs amongst health practitioners. Bartnik et al. (59), identified low levels of REDs awareness among general practitioners (10%) and physiotherapists (18%), suggesting that many primary-care providers remain insufficiently informed about REDs. Collectively, these findings suggest a persistent lag in the uptake of REDs terminology and concepts despite the increasing dissemination of updated guidelines. This limited uptake may contribute to delayed recognition and screening of individuals at risk of REDs (17, 60). However, these findings should not be interpreted as representative of all healthcare practitioners or clinical settings. Rather, they provide a historical snapshot of the challenges associated with disseminating and embedding updated conceptual frameworks within research.

Screening to identify REDs among at risk athletes is crucial to protect athlete health, wellbeing, and performance (1). This study found LEAF-Q was the most referred to validated screening tool, despite being validated only to female athletes, limiting identification of at-risk male and non-binary populations. Furthermore, the IOC's original Clinical Assessment Tool (CAT), introduced in 2015, was used infrequently. Among the four studies that cited the 2023 IOC Consensus Statement recommendations, remained limited with only one study reported using the updated CAT2 tool, a trend supported by a later, 2026 review by Muslichah et al. (61). This pattern may reflect a temporal lag between the publication of updated recommendations and their adoption within research design, compounded by the continued use of established assessment tools and frameworks (15, 22, 49). Notably, Heikura et al. (18), was recognised as the first study to begin application of the updated contemporary IOC 2023 recommendations, demonstrating that the updated tool can be successfully applied in research settings in a timely manner, even when minor methodological adaptations are required. As noted by Dvořáková et al. (62), the gradual incorporation of the CAT2 into published studies reflects methodological and timing-related factors rather than a lack of scientific acceptance or clinical relevance. Nevertheless, addressing this implementation gap remains critical for advancing early identification and standardised risk stratification in REDs research and clinical practice.

Following the dominant pattern of the Triad-focused research in this review, and despite evidence that LEA and REDs occur across sexes, performance levels, and sports (1, 11, 18, 34), athlete samples included in this study were represented by female, elite, collegiate, and endurance- or weight-sensitive sports. Male athletes, para-athletes, recreational participants, and team sport athlete samples were under-represented, limiting generalisability within the included studies. Broader sampling is needed to strengthen research of REDs across diverse population samples. This pattern is also consistent with previous reviews. Muslichah et al. (61) and Ghazzawi et al. (63), both emphasised the need for future research to move beyond the Female Athlete Triad framework and include athletes of all genders and from a broader range of sporting contexts. The under-representation of these populations may reflect historical research priorities and delays in translating contemporary guidance into research practice, rather than true differences in REDs prevalence.

Challenges in disseminating updated REDs guidelines appear to be influenced by the temporal gap between guideline publication and their incorporation into research design, a pattern reflected throughout this review. Study design, data collection, publication timelines, and the availability of contemporary guidelines can all influence the terminology, screening tools, and frameworks used by researchers. Although most included studies were published after the introduction of the IOC REDs guidelines, changes in conceptualisation and methodological practice may take time to be reflected in the literature. This was illustrated by Meyer et al. (45), where, despite publication after the release of the 2023 IOC REDs framework, the authors reported that the CAT2 was not available during the study period and therefore applied an earlier assessment framework (45). Conversely, Amoruso and colleagues (41), framed their study around the Female Athlete Triad despite being published more than a decade after the introduction of REDs. Together, these examples demonstrate how temporal factors can influence the adoption of updated terminology and frameworks within research. Accordingly, findings relating to guideline uptake should be interpreted cautiously, as dissemination, implementation, publication, and indexing timelines may substantially affect observed patterns. These considerations highlight that researchers are often constrained by the consensus frameworks available at the time a study is designed and conducted, rather than those available at the time of publication (58).

Within the included studies, persistent gaps remained between IOC guidelines and how REDs-related guidance and tools were operationalised in research that followed REDs or Triad concepts. Inconsistent terminology using terms interchangeable or in parallel, and low awareness, were commonly reported within the represented research. Encouragingly, studies describe incorporation of the contemporary IOC REDs guidelines, though, most use parallel with Triad concept demonstrating room for improvement within research and education contexts.

To improve utilisation of REDs frameworks and contemporary guidance within research, knowledge-translation efforts must more effectively align emerging evidence, conceptual models, and methodological design. Future research should prioritise timely translation of emerging IOC REDs guidance into study design to support strengthened methodologically aligned REDs research moving forward.

This review has several limitations. Consistent with scoping review methodology, no critical appraisal was undertaken; the findings should be interpreted as a map of how REDs-related guidance has been represented and operationalised in the literature, rather than an assessment of the quality or effectiveness of its implementation. Restricting inclusion to English-language publications may have introduced language bias. A single reviewer extracted data using predefined charting forms to support consistency, though this may still increase the risk of extraction bias. Because eligibility required explicit citation of REDs/RED-S/Triad/LEA guidance, the evidence base is inherently skewed toward research already aligned with these frameworks; this likely inflates the apparent prevalence of guideline use and cannot estimate real-world uptake, non-adoption, or clinical implementation. Non-reporting of referral pathways, multidisciplinary roles, or guideline use reflects reporting gaps rather than actual practice and should not be interpreted as evidence of non-implementation. Finally, given the typical lag between research evidence and clinical adoption, findings should not be taken as direct indicators of current practice. Temporal comparisons warrant particular caution, as absence of guideline citation in recent publications may reflect dissemination delays or study-period constraints rather than non-adherence.

## 5 Conclusion

This scoping review mapped how REDs-related guidelines, terminology, and screening tools were described within the published literature. Despite more than a decade of IOC consensus statements, the terminology remains inconsistent, validated screening tools are underutilised, and studies involving healthcare professionals report variable awareness and confidence in recognising REDs. The review also identified temporal factors that may influence the adoption of updated frameworks, terminology, and assessment tools within research. As studies are necessarily constrained by the guidelines and resources available at the time of their design and data collection, interpretations of terminology use and framework application should be made cautiously. Apparent inconsistencies or limited uptake may, in some cases, reflect publication, dissemination, and implementation timelines rather than conceptual misunderstanding. Collectively, these findings highlight the importance of the timely dissemination and integration of evolving REDs guidance within research to support athlete health, wellbeing, and performance.
