# Supplementary material for: Application of relative energy deficiency in sport (REDs) guidelines: a scoping review
Source: Front Sports Act Living. 2026 Oct 19;8:1932921. doi: 10.3389/fspor.2026.1932921 (PMC13725879; doi:10.3389/fspor.2026.1932921)
Supplement: Supplementary file 2 [file Supplementaryfile1.docx]

| **Supplementary File 1.**  **Evolution of Terminology Related to Low Energy Availability (LEA) and Relative Energy Deficiency in Sport (REDs)** | | | | | |
| --- | --- | --- | --- | --- | --- |
| **Term** | **Year Introduced / Updated** | **Defining Body** | **Definition** | **Key Contribution** | **Key Citation(s)** |
| **Low Energy Availability (LEA)** | **1990’s** | A. Loucks and Colleagues | EA=(EI−EEE). Threshold for clinical harm historically set at ≤30 kcal/kg FFM/day. | Identified as the aetiological factor underpinning REDs. Defined as a mismatch between dietary energy intake and exercise expenditure, leaving inadequate energy for body functions. | (1) |
| **Female Athlete Triad (Triad)** | **1980’s/**  **1992/ 2007** | B Drinkwater and American College of Sports Medicine (ACSM) | The combination of disordered eating, amenorrhea, and osteoporosis (1992). Redefined (2007) as a spectrum of relationships among energy availability, menstrual function, and bone mineral density. | Recognised the interrelationship between nutrition, reproductive health, and bone health in females. Introduced the concept of clinical spectrums ranging from health to disease. | (2-4) |
| **Relative Energy Deficiency in Sport (RED-S)** | **2014** | International Olympic Committee (IOC) | Impaired physiological function including metabolic rate, menstrual function, bone health, immunity, protein synthesis, and cardiovascular health caused by relative energy deficiency. | Expanded the 'Triad' concept to include male athletes and additional physiological systems (immunity, cardiovascular, psychological). Introduced the 'Sport Risk Assessment and Return to Play Model'. | (5) |
| **Relative Energy Deficiency in Sport (RED-S)** | **2018** | International Olympic Committee (IOC) | Syndrome of impaired physiological functioning caused by relative energy deficiency. Confirmed that optimal EA is ∼45 kcal/kg FFM/day, while systems are perturbed at <30 kcal/kg FFM/day. | Updated scientific progress on male athletes, para-athletes, and screening tools. Introduced the RED-S Clinical Assessment Tool (RED-S CAT) to assist in return-to-play decisions. | (6) |
| **Relative Energy Deficiency in Sport (REDs)** | **2023** | International Olympic Committee (IOC) | A syndrome of impaired physiological and/or psychological functioning caused by exposure to problematic (prolonged and/or severe) LEA. | Acronym updated to 'REDs'. Introduced a novel Physiological Model (adaptable vs. problematic LEA) and the REDs Clinical Assessment Tool-Version 2 (REDs CAT2) with a 4-color traffic light risk stratification. | (7) |
| **Note:** EA = Energy Availability; EI = Energy Intake; EEE = Exercise Energy Expenditure; FFM = Fat‑Free Mass; kg = kilogram; kcal = kilocalories.  **Reference List:** | | | | | |

1.	Loucks AB, Heath E. Dietary restriction reduces luteinizing hormone (LH) pulse frequency during waking hours and increases LH pulse amplitude during sleep in young menstruating women. The Journal of Clinical Endocrinology & Metabolism 1994;78(4):910-5. doi:[10.1210/jcem.78.4.8157720]

2.	Drinkwater BL, Nilson K, Ott S, Chesnut CH. Bone mineral density after resumption of menses in amenorrheic athletes. JAMA 1986;256(3):380-2.

3.	Nattiv A, Loucks AB, Manore MM, Sanborn CF, Sundgot-Borgen J, Warren MP. American College of Sports Medicine position stand. The female athlete triad. Medicine and science in sports and exercise 2007;39(10):1867-82. doi:[10.1249/mss.0b013e318149f111]

4.	Yeager KK, Agostini R, Nattiv A, Drinkwater B. The female athlete triad: disordered eating, amenorrhea, osteoporosis. Medicine & Science in Sports & Exercise 1993;

5.	Mountjoy M, Sundgot-Borgen J, Burke L, Carter S, Constantini N, Lebrun C, et al. The IOC consensus statement: beyond the female athlete triad—relative energy deficiency in sport (RED-S). British Journal of Sports Medicine 2014;48(7):491-7. doi:[10.1136/bjsports-2014-093502]

6.	Mountjoy M, Sundgot-Borgen J, Burke L, Ackerman KE, Blauwet C, Constantini N, et al. International Olympic Committee (IOC) consensus statement on relative energy deficiency in sport (RED-S): 2018 update. International Journal of Sport Nutrition and Exercise Metabolism 2018;28(4):316-31. doi:[10.1123/ijsnem.2018-0136]

7.	Mountjoy M, Ackerman KE, Bailey DM, Burke LM, Constantini N, Hackney AC, et al. 2023 International Olympic Committee’s (IOC) consensus statement on relative energy deficiency in sport (REDs). British Journal of Sports Medicine 2023;57(17):1073-97. doi:[10.1136/bjsports-2023-106994]
