# Supplementary material for: Application of relative energy deficiency in sport (REDs) guidelines: a scoping review
Source: Front Sports Act Living. 2026 Oct 19;8:1932921. doi: 10.3389/fspor.2026.1932921 (PMC13725879; doi:10.3389/fspor.2026.1932921)
Supplement: Supplementary file 3 [file Supplementaryfile3.docx]

| **Supplementary File 3.**  **Search strategy for Medline, CINAHL, SPORTDiscus and Scopus databases** | | |
| --- | --- | --- |
| 1 | MH | "Relative energy deficiency in sport" |
| 2 | Subject term | "Relative energy deficiency in sport" |
| 3 | Keywords | "Relative energy deficiency in sport" OR "REDs" OR "RED-s" OR "Low energy availability" OR "LEA" |
| 4 | Subject term | Interdisciplin* |
| 5 | Keywords | Interdisciplin* OR Multidisciplin* OR "health professional*" OR "health practitioner*" OR "health specialist*" OR Podiatr* OR Dietician OR nutritionist OR "Sport* Physician" OR Endocrinologist OR Physiotherap* |
| 6 | Keywords | Guidelin* OR recommen* OR Asses* OR Tool* OR Screen* OR Treat* OR Prevent* |
| 7 | Combine | 1 OR 2 OR 3 |
| 8 | Combine | 4 OR 5 |
| 9 | Combine | 6 AND 7 |
| 10 | Combine | 7 AND 8 AND 9 |
