# Supplementary material for: Application of relative energy deficiency in sport (REDs) guidelines: a scoping review
Source: Front Sports Act Living. 2026 Oct 19;8:1932921. doi: 10.3389/fspor.2026.1932921 (PMC13725879; doi:10.3389/fspor.2026.1932921)
Supplement: Supplementary file 4 [file Supplementaryfile4.docx]

| **Supplementary File 4. Data Extraction** | | | | | | |
| --- | --- | --- | --- | --- | --- | --- |
| **Health Professional Samples** | | | | | | |
| **Author Citation** | **Aim** | **Study Design**  **Participants**  **Gender** | **Guideline used tools** | **Guideline/Framework application** | **Applied by who** | **Competency** |
| **(1)** | The aim of the study was to assess the awareness of both the Triad and RED-S among multidisciplinary physicians, and to determine if an educational handout can improve awareness of the Triad and RED-S on a follow up survey. | Quantitative Survey Design  N=161 physicians completed the initial survey  N=49 Physicians completed the Follow-up survey  Multidisciplinary   - Emergency Medicine - Family Medicine, - Orthopaedic Medicine - Internal Medicine, - Physical Medicine - Rehabilitation and Primary Care Sports Medicine.   Gender nor Ethnicity not reported | Yes | 2018 Triad and RED-S consensus statements  Referenced IOC Relative Energy Deficiency in Sport Clinical Assessment Tool (RED-S CAT)  Educational handouts were given to recognising, diagnosing and treating athletes with LEA. (Mountjoy et al. 2014, 2015 & De Souza 2014)  Reassessed 5 weeks post handout | Multidisciplinary approach   - Physicians | Various levels of knowledge  Initial survey (n=161), 48% of physicians correctly identified the three criteria of the Triad, 54% correctly identified the three Triad criteria. and 32% correctly identified the key RED-S features follow up 40% correctly identified features of RED-S.  Among those who completed both surveys:  ▪ Triad recognition improved from 54% to 70% (n=46)  ▪RED-S recognition improved from 40% to 63% (n=43)  Orthopaedic surgeons showed the highest awareness (100% for both Triad and RED-S) |
| **(2)** | The aim of the study was to investigate the awareness and knowledge of physiotherapists, general practitioners, gynaecologists, sports physicians and orthopaedic surgeons on RED‐S. | Cross-sectional Design / online questionnaire  N=799  M: F = 240:559  ▪ Physiotherapists189: 70:119  ▪ General practitioners200: 53:147  ▪ Gynaecologists 281: 46:235  ▪ Orthopaedic surgeons43: 30:13  ▪ Sports physicians 86: 41:45  Ethnicity: Not reported, study conducted in the Netherlands | Yes | Recommendations on RED‐S from the IOC (Mountjoy et al., 2014) | Multidisciplinary team approach for treatment  According to the respondents, specialists who should be involved in the treatment of RED‐S include:   - Sports physicians (68.8%) - Sports dietitians (63.9%) - Sports Psychologists (49.6%), - Sports Physiotherapists (29.3%) - General practitioners (18.1%)   discrepancy in perceived involvement, 43.5% of physiotherapists felt physiotherapists should be involved in treating RED-S, compared to 4.8% of sports physicians who don’t think physio should be involved | Familiarity with RED-S   - Sports physicians 92.9%. - Orthopaedic Surgeon 23.3% - Physio 18.5% - Gynaecologist 11.0% - GPs 10.1%.   Heard of RED-S   - Only 22% of nearly 800 practitioners…   Competent discussing RED-S   - 57.6% reported   Treating RED-S   - 27.7% do not feel qualified - 7.4% felt qualified |
| **(3)** | The aim of the study was to assess awareness and comfort in treating patients with Triad and RED-S among providers attending a sports medicine conference. | Cross-sectional Design Survey  N= 163  M: F: Trans: T 74:88:1   - Physicians - Physician Assistants - Physical Therapists - Nurse Practitioners - Athletic Trainers - Allied health professional     Ethnicity: Not reported. Study set in Cambridge, Massachusetts, | Yes | Position statements on the Triad (American College of Sports Medicine and the National Athletic Trainers’ Association)  Refers to RED‐S of the IOC 2018  (Mountjoy et al., 2018) | Sports health professionals   - Was not further defined | Awareness   - TRIAD 76% - RED-S 29%   Felt Comfortable treating athletes   - Triad 33% - RED-S 13%   Heard of RED-S   - Physicians 36% - Non-physicians 24%   Fellowship trained physicians more aware   - Triad (91%) - RED-S (61%) - without fellowship training (63% and 23%)   Comfort treating athletes Triad   - Fellowship 57% - without Fellowship 26% - However, there was no significant difference in the level of comfort treating RED-S between fellowship-trained and non-fellowship-trained physicians |
| **(4)** | The aim of the study was to describe current methods of treatment and prevention used by physical therapists in the United States (US) for the female athlete triad. | Descriptive survey Analysis  N= 205  M: F102: 103  Physical Therapists  Ethnicity: Not reported, assumed American | YES | Pre-participation screening examinations (PPEs) monitoring female athletes  Strategies endorsed by the   - International Olympic Committee’s Medical Commission (IOCMC), - American College of Sports Medicine (ACSM) (Loucks et al., 2007), - National Athletic Trainer’s Association (NATA) (Bonci et al., 2008) | Physical Therapists - Athletic trainers may also screen for triad symptoms during pre-participation physical examinations  Referrals included:   - Physician - Registered Dietitian - Athletes Coach - Mental Health Professional - Sports Psychologist - Athletic Trainer   Multidisciplinary team approach is required for successful treatment and prevention | Triad accuracy   - 61% (126/205) of physical therapists surveyed stated they knew the three components of the female athlete triad - However, only 21% all three components   Specific Tx of TRIAD   - 26% (54/205) - Of this group, 24% (13 out of 54) assisted in athletic screening for the triad disorders during pre-participation physical examinations (PPEs)13. - 20% (11 out of 54) participated in PPEs but did not screen for triad disorders3. - More than half (52% or 28 out of 54) of the physical therapists who treated triad-related conditions did not participate in PPEs at all3. - 48% (26 out of 54) of physical therapists who had treated female athletes for triad disorders in the last 12 months, said they "always" (26%) or "sometimes" (22%) incorporate prevention strategies other than preseason screening   Screening Tools TRIAD   - 13/54 (24%)   Prevention Strategy TRIAD   - 48% (26/54)   Years of Experience   - 57% 16 years or greater - 22% 11–15 years - 17% 6–10 years - 4% 1–5 years   (59%) felt comfortable discussing disordered eating and menstrual dysfunction with female athletes  84% of respondents agreed that absent or abnormal menstruation is not a normal consequence of exercise |
| **(5)** | The aim of the study was to investigate the knowledge and management of RED-S, from the perspective of lightweight rowers and physiotherapists who have worked in lightweight rowing | Qualitative Interview Study  N= 12  M: F 6:6  Physiotherapists  Ethnicity: Not reported in the provided excerpts. | Yes | - RED-S Clinical Assessment Tool (CAT) Assessment Model (Mountjoy et al., 2015) - RED-S Return to Play (RTP) (Mountjoy et al., 2015). | Physiotherapist  Referrals included   - GP - Other Health Care Providers   Multi-disciplinary team involved   - sport psychologist, - gynaecologist, - endocrinologist, - sports dietitian, - sports medicine consultant | Knowledge of RED-S   - 42% of Physios - 5 physiotherapists (42%) knew of RED-S characteristics, while 7 (58%) had no knowledge of RED-S prior - Physiotherapists felt they lacked the necessary skillsets to recognise - Did not feel comfortable education or management. - physiotherapists in the study did not report applying specific guidelines for RED-S. The study found inappropriate management of RED-S was common due to a lack of knowledge - misconception among physiotherapists was that RED-S only affected women and their menstrual cycle |
| **(6)** | The aim of the study was to develop and validate a questionnaire to assess knowledge of the signs and symptoms of RED-S among two groups: health professionals and physically active individuals. | Cross-sectional study design  N = 97 (HP)  M: F 17:80   - physiotherapists, - dietitians, - general practitioners, - personal trainers - nurses,   NZ European, Māori, Pacific, Asian | Yes | - RED-S Knowledge Questionnaire (RED-SKQ) (Pai 2022) | Health Care Professionals | Health professionals had more knowledge than athletes |
| **(7)** | The aim of the study was to explore the perspective of athletes, coaches, and medical professionals on RED-S | Qualitative Interview Design  N= 4  M: F 1:3  Medical professionals.   - 2 Physio - 2 Doctor   Ethnicity: Not reported | No | - All coaches and medical personnel believed screening for risk factors associated with LEA and RED-S was necessary - medical personnel had not seen the RED-S Clinical Assessment Tool (CAT) before the interview, | Multidisciplinary Team involved   - The MDT includes players, coaches, medical personnel, and nutritionists, among others - for screening and management | 3 were able to identify an athlete with REDs  3 able to awareness of the female athlete triad and RED-S  All unaware of the RED-s CAT Tool |
| **(8)** | The aim of the study was to characterise the assessment and management practices employed by Sports Dietitians when assessing and managing athletes at risk of low energy availability (LEA). | Cross-sectional design  N= 55  M: F 5:50  Sports Dietitians  Ethnicity not provided but assumed Australian | Yes | - foundational guideline: Australian Institute of Sport RED-S Athlete Management Pathway Infographic (www.ais.gov.au) Australian Institute of Sport - Refers to the International Olympic Committee (IOC) consensus statement on relative energy deficiency in sport (RED-S): 2018 update - Low Energy Availability in Females Questionnaire (LEAF-Q) (Melin A 2014) | Sports Dietician  Referrals included   - sports physician for LEA management - Coach - Psychologist   Multidisciplinary interaction to manage LEA   - Physio, Psychologist, Physician, Sport Scientist, Coach, Manager, General Practitioner | Respondents rated the confidence in their ability to correctly assess an athlete's risk of LEA as 7.4 ± 1.3 on a 10-point scale.  Less than half of respondents 'always/often' use the Low Energy Availability in Females Questionnaire (LEAF-Q) (44.9%), other pathology (e.g., IGF-1, insulin, iron) (42.9%), or sex hormone pathology (LH, FSH, Testosterone) (46.9%) |
| **(9)** | The aim of the study was to assess the knowledge of collegiate ATs about the Triad and RED-S and to examine variability in related screening and referral behaviours among National Collegiate Athletic Association divisions. | Cross-sectional study.  N= 285  M: F 125:102 (58 genders not specified)  Head Athletic Trainers  Assume American otherwise not mentioned  The study noted that differences in knowledge between male and female ATs might exist, potentially rooted in sex-specific experiences, such as working with female athletes or personal experiences as athletes | Yes | Annual Preparticipation Examination and Screening occurring (Mountjoy 2014 & Nattiv et al, 2007)  International Olympic Committee (IOC) consensus statement on relative energy deficiency in sport (RED-S): 2014 | Athletic Trainers  Referrals included   - sports medicine physician - counsellor or psychologist - Dietician or Nutritionist | Respondents, 98.61% had heard of the Triad. (the 1% who didn’t know were men). Only 13.33% correctly identified energy imbalance as a component  32.98% had heard of RED-S  There was no significant difference between men and women ATs in having heard of RED-S  More female correctly identified the components of the condition. Women ATs correctly identified more components of the Triad than men ATs (U = 12.426, P = .003)  Experience  30.88% >20 years  31.23% 11-20 years  14.39% 6-10 years |
| **Note:** n = Number of participants; M = Male; F = Female; T = Total; N/R = Not reported; RED‑S = Relative Energy Deficiency in Sport; LEA = Low Energy Availability; IOC = International Olympic Committee; CAT = Clinical Assessment Tool; CAT2 = Clinical Assessment Tool Version 2; RTP = Return to Play; PPE = Pre‑participation examination; GP = General practitioner; Physio = Physiotherapist; HP = Health professional; AT = Athletic trainer; MDT = Multidisciplinary team. | | | | | | |

| **Athlete Samples** | | | | | | | |
| --- | --- | --- | --- | --- | --- | --- | --- |
| **Author** | **Aim** | **Study Design**  **Participants** | **Health Care Provider** | **Guidelines**  **used tools**  **Yes:No** | **Guideline application** | **Referral**  **Yes:No** | **Who to**  **What for** |
| **(5)** | The aim of this research was to investigate the knowledge and management of RED-S, from the perspective of lightweight rowers and physiotherapists who have worked in lightweight rowing. | Qualitative Interview Design  N= 12  M: F 4 (33.3%): 8 (66.67%)  BMI: N/R  Age: 22 (range 19 – 32)  Sporting Code: Lightweight Rowers  Level: Elite/ Olympic/ International  County of Population Ethnicity: | Physiotherapist | N | Recommended Tools: to inform medical management:   - RED-S Clinical Assessment Tool (CAT) Assessment Model (Mountjoy et al., 2015) - RED-S Return to Play (RTP) (Mountjoy et al., 2015). | Y | GP (Doctor)   - second opinion as the physio didn’t know why unwell rower   Other HCPs   - RED-S treatment.   However, discusses:  RED-S requires a multi-disciplinary team   - (e.g. sport psychologist, gynaecologist, endocrinologist, sports dietitian, sports medicine consultant). |
| **(6)** | The aim of this study was to develop and validate a questionnaire assessing knowledge of signs and symptoms of relative energy deficiency in sport among healthcare professionals and physically active individuals (PAI). | Cross-sectional study Design  N= 77  M: F 8: 66  BMI: N/R  Age: 31.5±12.5  Sporting Code: PAI  Level: N/R  New Zealand European | Health Professionals used to validate the screening:  physiotherapists,  dietitians,  general practitioners,  personal trainers,  nurses, | Y | Suggests RED-S Knowledge Questionnaire (RED-SKQ) is a validated screening tool  It specifically references the IOC consensus statement on RED-S (2014, 2018 update | N | Not reported |
| **(7)** | The aim of this study was to explore the awareness, risk factors, and strategies associated with RED-S amongst players, coaches, and medical professionals at a single Super League netball club. | Qualitative interview Design  N= 13  M: F 0:13  BMI: N/R  Age: 14 +  Sporting Code: Professional Netball  Level: Elite  UK | Medical Professionals  2 GP  2 Physio | N | No specific guidelines, protocols, or prevention tools were applied.  Gathered perspectives on potential applications and existing practice  Explored awareness of tools   - RED-S Clinical Assessment Tool (CAT)   Discussed strategies suggested by participants to mitigate RED-S risk, such as education, multidisciplinary team involvement | Y | Study discussed that a multidisciplinary team (MDT) approach involving players, coaches, medical personnel, and nutritionists should be responsible for RED-S screening and diagnosis |
| **(10)** | The aim of this study was to gain more insight into the reasons why female athletes do not seek help when experiencing amenorrhea and how care for these women could be improved. | Qualitative interview Design  N= 12  M: F 0:12  BMI: N/R  Age: 33 years (range 23—45).  Sporting Code: cycling, speed  skating, athletics, running, triathlon and mountain biking  Level: ex elite or professional  Dutch | Coaches, trainers, physiotherapists, and general practitioners. | N | Signalling role (Mountjoy 2014)   - *discussed by* the participants or *recommendations derived from* the study's findings for future care, rather than guidelines directly applied *to* the female athletes | Y | Importance of a multidisciplinary collaboration of RED-S for optimisation of care to address the various aspects of Relative Energy Deficiency in Sport (RED-S) Suggesting referrals or involvement of different   - GPs should be informed about menstrual problems - specialisms and paramedics, including - gynaecologists, - sports physicians, - sports dieticians, and psychologists, |
| **(11)** | The aim of this study was to address these gaps by  evaluating the impact of a nutrition education intervention led by a  registered dietitian on energy availability, anthropometric  measurements, sports nutrition knowledge, and eating attitudes in adolescent female team sport athletes | RCT  N= 83  M: F 0:83  BMI: 20.68 ± 2.28 kg/m2  Age: aged 15 & 18 (mean 17.2 ± 2.0)  Sporting Code: football, basketball, and volleyball  Level: Elite  Control group: n=38  Intervention group: n=45 | Registered dietitian | Y | - (LEAF-Q) (Melin 2014), - (EAT-26) (Mintz 2000), - (SNKQ) (Zinn C 2005). - Energy and nutrient intakes were evaluated through 3-day food records (ACSM Thomas 2016), - Anthropometric Measurements: - Exercise energy expenditure was assessed using 3-day activity logs. (Heikura IA, 2017)   Energy Availability (EA):  Resting Metabolic Rate (RMR):  Exercise Energy Expenditure (EEE):  Intervention Protocol (Nutrition Education): Six physical face-to-face sessions | Y | Dietician   - nutrition education - Disorder eating/ eating disorder |
| **(12)** | The aim of this study was to translate, culturally adapt, and validate the Brazilian version of LEAF-Q as an instrument to identify athletes at risk for the Triad based on the analysis of reliability and construct validity by known-groups or contrasting groups method. | Second stage of the study: Test-Retest Reliability Analysis and Construct Validation  N= 127  M: F 0:127  BMI: mean 23.4 (2.8)  Age: mean 27 (6.4)  Sporting Code: running, triathlon, swimming, cycling, rhythmic gymnastics, jiu-jitsu, Muay Thai, mixed martial arts (MMA), rugby, football, basketball, volleyball, handball, and CrossFit®  Level: N/R  Brazilian Athletes | psychology, nutrition, physical education, and  Medicine | Y | Low Energy Availability in Females  Questionnaire (LEAF-Q) (Melin 2015) | N | Not reported |
| **(13)** | The aim of this study was to assess the energy availability (EA) of Paralympic track and field athletes (sprinters). | Pioneer study. Cross-sectional design.  N= 17  M: F 9:8  BMI: mean 61.93  Age: mean 25.6  Sporting Code: Sprinters  Level: Paralympic  Brazilian | Professional team  dietitian,  physician,  psychologist and  physiotherapist | Y | Physical evaluation   - three monthly/ 1 year. - extensive routine of physical tests/assessments - EA Values   *uses* established EA cut-off values (e.g., ≤ 30 kcal/kgFFM/day for LEA and ≥ 45 kcal/kgFFM/day for adequate EA) | N | Not reported |
| **(14)** | The aim of this study was to establish a baseline understanding of dance students at a single pre-professional institution based on metrics focused on current health, nutrition, lifestyle, and wellbeing while also gauging knowledge of longer-term health implications | Cross-sectional study design  N= 63  M: F 5:58  BMI: mean 20.5 kg/m2. Females 47% < 20kg/m2  Age: mean 19.24 years  Sporting Code: Dance  Level: pre-professional  Australian | Healthcare practitioners | Y | Dance-Specific Energy Availability Questionnaire (DEAQ) Keay et al.  Draws on three validated questionnaires,   - (LEAF-Q), - Sports Specific Energy Availability Questionnaire (SEAQ-I), - (REDs CAT) - Australian Guide to Healthy Eating   Recommends   - Screened for LEA and REDs on admission and annually as a general health check (Paschali 2023) - Dance health Training REDs Health Conceptual Model and REDs Performance Conceptual Model Mountjoy et al. 2023 - Screening questions physical and health screenings (Matiussi 2023) | Y | Recommends qualified sports dietitian Civil et al 2018.   - enhance knowledge and personalise advice that is safe and sustainable.   Recommends Multidisciplinary, collaborative approach. Reece et al.2023   - disordered eating/eating disorders |
| **(15)** | The aim of this study was to conduct nutritional (3-day dietary diaries) and body composition (ISAK profile) assessments on sport climbing athletes by gender and climbing level | Cross-sectional study design  N= 45  M: F 22:23  BMI: 21.02kg/m2  Age: mean 30 years  Sporting Code: Sport climbers  Level: Experienced  Spanish | Not reported | Y | Gold standard for an adequate nutritional or dietary assessment Burke, 2015.  Nutritional Assessment Beck 2021  EA=(EI −EEE)/FFM  Sports characteristics questionnaire (   - [Compendium of Physical Activities](https://pacompendium.com/) Quantifying Physical Activity Energy Expenditure Ainsworth, B 2011) | N | Not reported |
| **(16)** | The aims of this study was to  examine associations between EA/MD and energy  metabolism and the prevalence of Triad-associated conditions in endurance athletes. | Observational Study  N= 40  M: F 0:40  BMI: 20.5±1.9  Age: 18-38 years Mean 26.3±5.7  Sporting Code: Endurance Sport  Level: Elite & Competitive | Dietician  Gynaecologist | Y | EA Assessment Loucks et al., 2011   - nutrient analysis program - assess exercise energy expenditure (EEE) Tomten & Hostmark, 2006) - Eating Disorder Inventory (EDI-3), questionnaire (Garner 2004) - Eating Disorder Examination (EDE-16) (Cooper et al., 1989), - DXA, dual-energy X-ray absorptiometry. (Nattiv et al., 2007)) - RMR, resting metabolic rate - Gynaecological assessment. - Blood Sampling - Anamnestic assessment - low EA in females’ questionnaire (LEAF-Q) (Melin et al., 2014). | N | However, mentions:   - The findings of either one of these related conditions, therefore, should implicate a careful and expanded assessment. Mountjoy 2014 - Prevention, early detection, and treatment of energy deficiency and MD is important to prevent health consequences and injuries, but also to optimize performance and recovery, (IOC Consensus Statement 2014) |
| **(17)** | The aim of this study was to describe dietary characteristics of athletes with LEA and/or FHA. | Cross-sectional study  N= 25  M: F 0:25  BMI: 20.6±2.0  Age: 26.6±5.6  Sporting Code: Endurance sports  Level: Elite  Scandinavian | Dietician  Gynaecologist | Y | Protocols included  Eating disorder evaluation,   - 7-day dietary intake and EA assessment (Loucks, 2014) - Eating Disorder Inventory (EDI-3; Garner, 2004)   Anthropometric assessment   - body weight and height were measured - dual-energy X-ray absorptiometry (DXA) Nattiv 2007   Examinations of reproductive function.   - trans vaginal ultrasound examination, - sex hormone status, - the Low EA in Females Questionnaire (Melin et al., 2014b). | Y | Multidisciplinary professional   - Dietician - Professional counselling   Address the   - Underlying psychological factors (Nattiv et al., 2007; Mountjoy et al., 2014). - Endocrine and reproductive disruptions - Modifying the diet and/or the training regime, (De Souza et al., 2014). - Dietary recommendations increasing energy intake with 300–600 kcal (1.2–2.4 MJ)/day (Mountjoy et al., 2014) - Early detection and intervention of LEA and FHA to prevent long-term health consequences (Nattiv et al., 2007; De Souza et al., 2014; Mountjoy et al., 2014). |
| **(18)** | The aim of this study was to develop and test a screening tool designed to identify female athletes at risk for the Triad. | Observational study design  Clinical verification/ validation  N= 45  M: F 0:45  BMI: 20.5±1.8  Age: 18-39 years 26.6±5.4  Sporting Code: Dance and Endurance  Level: N/R  Scandinavian | endocrinology,  sports nutrition,  medicine and gastroenterology | Y | - Low EA in females’ questionnaire (LEAF-Q) (Melin et al., 2014). - Eating Disorder Inventory (EDI-3) Garner, 2004) - Eating Dis order Examination (EDE-16.0; Fairburn et al., 2008), - Dietary intake and training intensity to assess EA. Louks 2007 - Assess exercise energy expenditure (EEE) Tomten & Hostmark, 2006   Examinations of reproductive function.   - trans vaginal ultrasound examination, - sex hormone status, - Pregnancy Test   Anthropometric assessment  • body weight and height were measured  • dual-energy X-ray absorptiometry (DXA) Nattiv 2007 | Y | Further referral:   - Gynaecologist - implicate assessment for the other conditions, including a pathological eating behaviour. - Importance of differential diagnoses proper intervention |
| **(19)** | The aim of this study was to assess the prevalence of Triad and Red-S using the LEAF-Q in youth female football players. | Cross-sectional  N= 34  M: F 0:34  BMI: 20.16  Age: 13-18 years 15.41mean  Sporting Code: Football  Level: Youth  Polish | Medical Staff | Y | LEAF-Q questionnaire Melin 2014  Emphasizes the importance of early identification by screening tools like the LEAF-Q to protect young athletes from long-term damage  Dual energy X-ray absorptiometry Nattiv 2007  Energy intake (EI) collected | N | Not reported |
| **(20)** | The aims of this study was to (1) identify risk of LEA in females competing at different levels of competition and  (2) investigate associations between risk of LEA, illness and dietary habits. | Cross-sectional design.  N= 833  M: F 0:833  BMI: 23.62 kg/m2 (±4.26 kg/m2)]  Age: 18 years or older  Sporting Code: Individual & Team soccer, rugby, cricket, archery, volleyball, long-distance running/triathlon, hockey, cycling, and rowing  Level: Active – Elite  Irish | Online Questionnaire | Y | The LEAF-Q (Melin et al., 2014).  Mountjoy IOC  Not used in this study but refers to:   - Investigated biomarkers (Koehler et al., 2016; Lagowska & Kapczuk, 2016; Schaal, Tiollier, Le Meur, Casazza, & Hausswirth, 2016)   Athlete-specific ED questionnaires   - The Female Athlete Screening Tool (FAST) (McNulty, Adams, Ander son, & Affenito, 2001) - Brief ED in Athletes Questionnaire (BEDA-Q) (Martinsen, Holme, Pensgaard, Torstveit, & Sundgot-Borgen, 2014),   study identifies existing guidelines and recommendations (like the LEAF-Q screening tool and IOC consensus statements) but frames their comprehensive application and integration into routine practice | N | Sports Physician:   - Screening for risk of LEA - Document the injury and illness patterns   Clinical Evaluation:   - menstrual issues/ dysfunction, polycystic ovary syndrome (PCOS), Gordon et al., 2017   This study encourages:   - Regular screening (Mountjoy et al., 2015), - Educational interventions   study suggests that athletes, coaches, and parents should inform themselves about psychological and physiological changes |
| **(21)** | The aim of this study was to estimate the prevalence of European cross-country athletes at risk of LEA using the LEAF-Q and to examine demographic and physical characteristics that are associated with risk of LEA. | Cross-sectional Design  N= 207  M: F 124:83  BMI: 19.3  Age:18-35 years mean 22.1  Sporting Code: Cross-Country  Level: Elite  Portugal(16.6%) Spain 12.1%), Italy(8.2%), Ireland(7.7%) ,and the United Kingdom (4.8%), among others. Participants were mostly Caucasian (89.4%,n=185), but also included Black (8.7%,n=18), Asian (1.4%,n=3), and Arabic(0.5%,n=1) athletes. | Self-administrated questionnaire | Y | Athlete’s risk assessments 1 – 3 monthly   - Low Energy Availability in Females Questionnaire (LEAF-Q) Melin’s 2014 - Low Energy Availability in Females Questionnaire (LEAF-Q), (exclude the menstrual section for males) Slater 2015   Refers to the   - RED-S model Mountjoy 2018 - the Female Athlete Triad model De Souza et al. | Y | Multidisciplinary team   - To prevent, educate, treat, or minimize LEA and its effects on the body physiological functioning. - Detailed body composition analysis (e.g., fat mass and FFM) and blood markers analysis   Follows Mountjoy 2018 RED-S Model   - identification of the risk factors for athletes at risk of LEA, - Prevent the development or to initiate the treatment of the consequences |
| **(22)** | This study aims to assess the dietary intake and energy consumption of non-professional student female dancers as a preliminary investigation into this topic. | Cross-sectional study Design  N= 25  M: F 0:25  BMI: 24.6 ± 1  Age: 21 ± 1  Sporting Code: Dance  Level: non-professional  Iranian | Self-administered  Dietician | Y | Self-reported food diaries 7-day period,   - Total Energy Expenditure (TEE), - Total Energy Intake (TEI), - macronutrient intake. - = EA - Average Daily Energy Calculation: - Energy Expenditure on Dance Days (EED) Adjustment: - Energy Expenditure on Rest Days (EER): - Energy Expenditure on Working Days (EEW): - Energy Needs for Dancing: | Y | Consulting experienced   - Sports nutritionists and other related specialists, including psychiatrists, - Developing strategies to increase nutritional literacy - Preventing these unhealthy nutritional behaviours. |
| **(23)** | This study aimed to investigate the physical and psychosocial impact of RED-S from the personal perspective of lightweight rowers. | Qualitative Interviews Study Design  N= 12  M: F 4:8  BMI: N/R  Age: mean 22 years (range 19 to 32)  Sporting Code: Lightweight Rowing  Level: Intermediate – Elite  United Kingdom | Physiotherapist | Y | Semi-structured individual qualitative interviews   - Assessing Short and long-term physical and psychosocial impacts   IOC Mountjoy 2014  It references the IOC consensus statement on Relative Energy Deficiency in Sport (RED-S) | Y | However, suggests:   - Need for effective strategies to prevent and manage RED-S - improving education and support for athletes. - importance of awareness and education of RED-S - RED-S prevention strategies may benefit from being sport-specific |
| **(24)** | This study aimed to clarify the relationship between the triad  risk assessment score and the sports injury rate | Cross Sectional Design  N= 116  M: F 0:116  BMI: N/R  Age: N/R  Sporting Code: swimming, athletics sprint, athletics long-distance, athletics throwing/jumping, soccer,  basketball and volleyball  Level: National  Ethnicity: Likely Japanese – as conducted at a Japan University. No specific mention | Self-report questionnaire form  physical therapists and a medical doctor | Y | Female Athlete Triad Coalition Consensus Statement   - Female Athlete Triad Cumulative Risk Assessment (De Souza et al., 2014). - BMD was measured (De Souza et al., 2014). - Injury Survey IOC 2008 - the six factors scored were: (1) LEA with or without disordered eating (DE)/eating disordered (ED); (2) low BMI; (3) delayed menarche; (4) oligomenorrhea and/or amenorrhea; (5) low BMD; and (6) stress reaction/fractures | Y | Medical doctor   - X-rays and MRI stress fracture |
| **(25)** | The aim of this study was to identify risk factors for ill ness athletes and quantify the relative importance of risk factors to inform the design of preventive measures for Olympic-level athlete cohorts. | Cross Sectional Study Design  N= 81  M: F 26:55  BMI: N/R  Age: M 25.4 ± 6.2: F 25.5 ± 7.4 years  Sporting Code: athletics, cycling, equestrian, gymnastics, hockey, rowing, rugby sevens, sailing; swimming, triathlon, water polo,  Level: Elite  Australian Olympic athletes | Clinicians | Y | Screening Tools and Questionnaires   - Dispositional Resilience Scale, Funk 1992 - Recovery-Stress Questionnaire (REST-Q-52 item), Kellmann 2001 - Low Energy Availability in Females Questionnaire (LEAF-Q), Melin 2017 - Epworth Sleepiness Scale, Johns 1991 - Pittsburgh Sleep Quality Index Buysse 1989 - The Personal and Household Hygiene questionnaire for university students Miko 2013 - Depression, Anxiety, Stress Scale (DASS-21) Antony 1998 Henery 2005 - Perceived Stress Scale, Cohen S 1983 | N | Prevention of Injury and Protection of Athlete Health supported by the International Olympic Committee (IOC).   - clinicians should be vigilant for declining mental health - importance of educating athletes and coaches - Illness prevention programmes should address energy availability, mental health, travel, living situations, hygiene and sex-specific issues. - Clinicians charged with the care of ill athletes might need to investigate the psychological, environmental, nutritional and hygiene components of the presentation. - Illness prevention programmes should evaluate the effect of multi-factorial preventive measures. |
| **(26)** | This study aimed to establish the prevalence of illness symptoms, poor sleep quality, poor mental health symptoms,  low energy availability and stress-recovery state in an  Olympic cohort late in the 3 months prior to the Summer  Olympic Games. | Cross Sectional Study Design  N= 132  M: F 47:85  BMI: N/R  Age: M 25.8±4.1; F 24.3±3.9 years  Sporting Code: boxing (n=1), equestrian (n=1), football (soccer) (n=20), gymnastics (n=1), hockey (n=43), rowing (n=13), rugby sevens (n=32), sailing (n=1), triathlon (n=2), water polo (n=15), and unreported (n=3)  Level: Elite Olympic  Australian | Questionnaires | Y | Periodical Health Examination. Drew 2017  Questionnaires included the   - Depression, Anxiety and Stress Questionnaire, Antony 1998 - Perceived Stress Scale, Cohen S 1983 - Dispositional Resilience Scale, Funk 1992 - Recovery-Stress Questionnaire (REST-Q-52 item), Kellmann 2001 - Low Energy Availability in Females Questionnaire (LEAF-Q), Melin 2017 - Epworth Sleepiness Scale, Johns 1991 - Pittsburgh Sleep Quality Index Buysse 1989 - Custom-made questionnaires on probiotic usage and travel. | Y | Support multidisciplinary care.   - Athlete health is best managed by a multidisciplinary team of medical and other practitioners. - Priority for multidisciplinary prevention and management programmes - Direct measurement of LEA should be included in prevention and management programmes - Individualised care is recommended. - Illness prevention programme includes evaluation of multifactorial preventive measures. - Injury Definitions Concept Framework Timpka T 2015 |
| **(27)** | This study aimed to determine bone mineral density (BMD)  and the associations among BMD, menstrual history,  disordered eating (DE), training history, intentional weight loss (IWL) and rib pain for the first time in female  lightweight rowers. | Cross Sectional Study Design  N= 21  M: F 0:21  BMI: 21.4±2.0  Age: 28.7±4.3  Sporting Code: Rowing - lightweight  Level: International/ National/ Elite  UK Caucasian | Multidisciplinary | Y | Investigate the distribution of bone mineral density (BMD)  Examined BMD and total body composition via   - Dual-energy X-ray absorptiometry, The ACSM Navitti 2007 - Menstrual history, Cobb et al 2003 - Disordered eating, EAT-26 Garner et al. 1989 - Training history, - Intentional weight loss - Rib pain | Y | Multidisciplinary sport science support   - support bone and general health, - injury prevention - sporting performance, - current training paradigms   Nutritional Guidance   - Prevention and treatment of DE Beals and Manore, DE preventative programme. Sundgot-Burgen and Klungland - Address athletes exercise energy requirements nutritional knowledge.   Psychologist Support and Assessment   - Assessment of DE - Identify psychopathological - Educate, prevent, recognise, support and manage weight loss and DE   Clinical investigation   - low BMD Z-score of < -1.0 in an athlete warrants further investigation and management |
| **(28)** | This study aimed to determine if following specific diets was associated with reporting behaviours that are consistent with disordered eating compared to non-diet-adherent athletes. | Cross Sectional Study Design  N= 931  M: F 0:931  BMI: N/R  Age: 18.92±3.34  Sporting Code: N/R  Level: Competitive | Not reported | Y | Eating disorder screening tools   - Brief Eating Disorder in Athletes Questionnaire (BEDA-Q) Martinsen 2014 - Eating Disorder Screen for Primary Care (ESP), Cotton 2003 | Y | Healthcare providers interdisciplinary approach   - Including nutritional, registered dietitian and psychological support. - Evaluate individuals’ nutritional knowledge, and monitoring of energy intake - Questionnaires - Educate athletes regarding macronutrient and micronutrient deficiencies - interdisciplinary approach focusing on patient-centred care |
| **(29)** | The aim of this study was to identify the possible role of menstrual irregularity alongside other behavioral, anthropometric, and sport-related parameters in a sample of Italian female athletes. | Cross Sectional Study Design  N= 288  M: F 0:288  BMI: N/R  Age: 15–49 years  Sporting Code: National sport federations  Level: National  Ethnicity: Italian | Multidisciplinary | Y | American College of Sports Medicine's description of the Female Athlete Triad (1992) serves as the foundational framework for the study  Original structured questionnaire composed by:   - Eating Disorder Examination Questionnaire (EDE-Q) (Aardoom et al., 2012); - Low En ergy Availability in Females Questionnaire (LEAF-Q) (Łuszczki et al., 2021);   Brief Eating Disorder in Athletes (BEDA-Q) (Martinsen et al., 2014). | Y | Multidisciplinary preventive approach.   - Screenings and awareness programs - Evaluation of menstrual regularity - Assessment of energy availability (e.g. TEE, lean and fat mass, BMC bone- fracture) - Testing for sport-derived stress and disordered eating attitudes - Periodic competitive sport medical assessment/examination. - Assessments questionnaire, addressing dual-energy X-ray absorptiometry (DXA) testing on participating athletes. |
| **(30)** | The aim was to determine the prevalence and awareness of low energy availability (LEA) using the LEAF-Q in female Gaelic Athletic Association (GAA) athletes | Cross-sectional cohort (survey) design (online survey) of club teams  N= 122  M:F 0:  Median age 22 (IQR = 9).  Mean BMI 24.55 kg/m² (± 4.94). Ireland.  Sporting codes: 77 (63 %) played Gaelic Football only, 23 (19 %) played Camogie only, and 22 (18 %) played both. | Not reported | Y | Low Energy Availability in Females Questionnaire (LEAF-Q) to screen for risk of LEA.  Consensus statements (e.g. IOC consensus on RED-S, Female Athlete Triad guidelines) | N | does *not* detail a formal referral pathway in its methods or results  need for “access to multidisciplinary supports,”  benefit from further evaluation or intervention (e.g. nutrition, medical, bone health), but that is proposed rather than executed. |
| **(31)** | To characterize athletes who present to an outpatient interdisciplinary / multidisciplinary consultation for suspected REDs (Relative Energy Deficiency in Sport), both cross-sectionally and longitudinally, and to evaluate the efficacy of this treatment approach. | Observational clinical practice (prospective / longitudinal observational)  58 athletes suspected of REDs  M:F 2:56  97 % were female  median age was 16.5 years (range 12.5–44.0)  (BMI < 18.5 kg/m²  Endurance sports and Aesthetic Sports | Multiple departments / specialties are involved: Sports Medicine, Gynaecology, Psychosomatic Medicine / Psychiatry, Dietary Counselling / Nutrition. | Y | They used the IOC Clinical Assessment Tool Version 1 (CAT1) as their risk assessment tool for REDs.  refer to the newer CAT2 model (four-level traffic light system) in discussion  They also refer to consensus statements (e.g. IOC consensus on REDS) for guidance. | Y | The clinic had an integrated referral / collaboration approach: patients seen initially in Sports Medicine; then referrals / shared care with Gynecology, Psychosomatic Medicine / Child & Youth Psychiatry, Dietary Counseling as needed.  Who: Athletes flagged as suspected REDs, especially those with underweight, menstrual disorders, weight loss, low body fat, or history of bone stress injuries.  What for: For comprehensive assessment and management — monitoring anthropometry and weight, nutritional intervention, psychological / eating disorder treatment, menstrual / gynecological evaluation, bone health assessment, adjustment of training / sport participation (e.g. partial or total sport restriction where needed). |
| **(32)** | To implement the 2023 IOC REDs CAT2 for the first time in a large cohort of elite athletes to determine the current severity and prevalence of REDs  To prospectively evaluate whether baseline CAT2 traffic-light severity/risk status (green, yellow, orange, or red light) predicts the future risk and severity of self-reported physician-diagnosed bone stress injuries over 6–24 months | Cross-sectional retrospective and prospective study  213 Elite Athletes  M:F 70:143  Females 23.3 ± 4.3 years; Males 23.1 ± 3.7 years  National to elite/world-class Canadian Olympic and Paralympic athletes | Sports medicine physicians, endocrinologists, physiologists, dietitians | Y | 2023 IOC REDs CAT2  LEAF-Q (Low Energy Availability in Females Questionnaire, derived/modified self-report online questionnaire for menstrual history) | N | The guideline framework was applied strictly for epidemiological risk stratification (traffic-light classification) and prospective BSI outcome prediction within a research setting, rather than active clinical intervention or referral management |
| **Note:** n = Number of participants; Y/N = Yes/No; N/R = Not reported; M = Male; F = Female; T = Total; BMI = Body mass index; RED‑S = Relative Energy Deficiency in Sport; LEA = Low Energy Availability; IOC = International Olympic Committee; CAT = Clinical Assessment Tool; CAT2 = Clinical Assessment Tool Version 2; RTP = Return to Play; PPE = Pre‑participation examination; GP = General practitioner; Physio = Physiotherapist; HP = Health professional; AT = Athletic trainer; MDT = Multidisciplinary team; PAI = Physically active individuals.  **References** | | | | | | | |

1.	Warrick A, Hassid B, Coleman B, Cansino C, Faustin M. Multidisciplinary physician survey assessing knowledge of the female athlete triad and relative energy deficiency in sport. Journal of Eating Disorders 2023;11(1):doi:[10.1186/s40337-023-00800-4]

2.	Verhoef S, Wielink M, Achterberg E, Bongers M, Goossens S. Knowledge on relative energy deficiency in sport among physiotherapists and physicians. European Journal of Sport Science 2024;24(1):156-63. doi:[10.1002/ejsc.12026]

3.	Tenforde A, Beauchesne A, Borg-Stein J, Hollander K, McInnis K, Kotler D, et al. Awareness and comfort treating the female athlete triad and relative energy deficency in sport among healthcare providers. Dtsch Z Sportmed 2020;71(3):69–76. doi:[10.5960/dzsm.2020.422]

4.	Pantano KJ. Strategies used by physical therapists in the U.S. for treatment and prevention of the female athlete triad. Physical therapy in sport : official journal of the Association of Chartered Physiotherapists in Sports Medicine 2009;10(1):3-11. doi:[10.1016/j.ptsp.2008.09.001]

5.	Gillbanks L, Mountjoy M, Filbay SR. Insufficient knowledge and inapproriate physiotherapy management of Relative Energy Deficiency in Sport (RED-S) in lightweight rowers. Physical Therapy in Sport 2022;54(8-15. doi:[10.1016/j.ptsp.2021.12.002]

6.	Pai N, Brown C, Black E. The development and validation of a questionnaire to assess relative energy deficiency in sport (RED-S) knowledge. Journal of Science and Medicine in Sport 2022;25(10):794-9. doi:[10.1016/j.jsams.2022.07.004]

7.	O'Donnell J, White C, Dobbin N. Perspectives on relative energy deficiency in sport (RED-S): A qualitative case study of athletes, coaches and medical professionals from a super league netball club. PLoS ONE 2023;18(5):doi:[10.1371/journal.pone.0285040]

8.	Bowler A-LM, Coffey VG, Cox GR. Sports Dietitian practices for assessing and managing athletes at risk of low energy availability (LEA). Journal of Science & Medicine in Sport 2022;25(6):460-5. doi:[10.1016/j.jsams.2022.02.002]

9.	Kroshus E, DeFreese JD, Kerr ZY. Collegiate athletic trainers' knowledge of the female athlete triad and relative energy deficiency in sport. Journal of Athletic Training 2018;53(1):51-9. doi:[10.4085/1062-6050-52.11.29]

10.	Verhoef SJ, Wielink MC, Achterberg EA, Bongers MY, Goossens SMTA. Absence of menstruation in female athletes: why they do not seek help. BMC Sports Science, Medicine and Rehabilitation 2021;13(1):doi:[10.1186/s13102-021-00372-3]

11.	Tektunalı Akman C, Gönen Aydın C, Ersoy G. The effect of nutrition education sessions on energy availability, body composition, eating attitude and sports nutrition knowledge in young female endurance athletes. Frontiers in Public Health 2024;12:(doi:[10.3389/fpubh.2024.1289448]

12.	Pereira de Maria U, Ridel Juzwiak C. Cultural Adaptation and Validation of the Low Energy Availability in Females Questionnaire (LEAF-Q). Rev Bras Med Esporte 2021;27(2):184-8. doi:[10.1590/1517-869220212702223889]

13.	Paduan Joaquim D, Ridel Juzwiak C, Winckler C. Do paralympic track and field athletes have low energy availability? Os atletas do atletismo paralímpico tem baixa energia disponível? 2018;20(1):71-81. doi:[10.5007/1980-0037.2018v20n1p71]

14.	Nicholas JG, S. Investigating pre-professional dancer health status and preventative health knowledge. Frontiers in Nutrition 2023;10:(doi:[10.3389/fnut.2023.1271362]

15.	Mora-Fernandez A, Argüello-Arbe A, Tojeiro-Iglesias A, Latorre JA, Conde-Pipó J, Mariscal-Arcas M. Nutritional Assessment, Body Composition, and Low Energy Availability in Sport Climbing Athletes of Different Genders and Categories: A Cross-Sectional Study. Nutrients 2024;16(17):doi:[10.3390/nu16172974]

16.	Melin A, Tornberg Å, Skouby S, Møller S, Sundgot-Borgen J, Faber J, et al. Energy availability and the female athlete triad in elite endurance athletes. Scandinavian Journal of Medicine & Science in Sports 2015;25(5):610-22. doi:[10.1111/sms.12261]

17.	Melin A, Tornberg Å, Skouby S, Møller S, Faber J, Sundgot‐Borgen J, et al. Low-energy density and high fiber intake are dietary concerns in female endurance athletes. Scandinavian Journal of Medicine & Science in Sports 2016;26(9):1060-71. doi:[10.1111/sms.12516]

18.	Melin A, Tornberg Å, Skouby S, Faber J, Ritz C, Sjödin A, et al. The LEAF questionnaire: a screening tool for the identification of female athletes at risk for the female athlete triad. British Journal of Sports Medicine 2014;48(7):540-5. doi:[10.1136/bjsports-2013-093240]

19.	Łuszczki E, Jagielski P, Bartosiewicz A, Kuchciak M, Dereń K, Stolarczyk A, et al. The LEAF questionnaire is a good screening tool for the identification of the Female Athlete Triad/Relative Energy Deficiency in Sport among young football players. PeerJ 2021;9:(e12118. doi:[10.7717/peerj.12118]

20.	Logue DM, Madigan SM, Heinen M, McDonnell S-J, Delahunt E, Corish CA. Screening for risk of low energy availability in athletic and recreationally active females in Ireland. European Journal of Sport Science 2019;19(1):112-22. doi:[10.1080/17461391.2018.1526973]

21.	Jesus F, Castela I, Silva AM, Branco PA, Sousa M. Risk of low energy availability among female and male elite runners competing at the 26th European cross-country championships. Nutrients 2021;13(3):1-11. doi:[10.3390/nu13030873]

22.	Hajian M, Mohaghegh S. Do Iranian Female Dancers Face a Risk of Chronic Negative Energy Balance? A Study on Energy Intake and Expenditure Among Non-professional Female Dancers. Asian Journal of Sports Medicine 2024;15(2):doi:[10.5812/asjsm-139119]

23.	Gillbanks L, Mountjoy M, Filbay SR. Lightweight rowers' perspectives of living with Relative Energy Deficiency in Sport (RED-S). PloS one 2022;17(3):e0265268. doi:[10.1371/journal.pone.0265268]

24.	Edama M, Inaba H, Hoshino F, Natsui S, Maruyama S, Omori G. The relationship between the female athlete triad and injury rates in collegiate female athletes. PeerJ 2021;9:(doi:[10.7717/peerj.11092]

25.	Drew MK, Vlahovich N, Hughes D, Appaneal R, Peterson K, Burke L, et al. A multifactorial evaluation of illness risk factors in athletes preparing for the Summer Olympic Games. Journal of Science & Medicine in Sport 2017;20(8):745-50. doi:[10.1016/j.jsams.2017.02.010]

26.	Drew M, Vlahovich N, Hughes D, Appaneal R, Burke LM, Lundy B, et al. Prevalence of illness, poor mental health and sleep quality and low energy availability prior to the 2016 Summer Olympic Games. British Journal of Sports Medicine 2018;52(1):47-53. doi:[10.1136/bjsports-2017-098208]

27.	Dimitriou L, Weiler R, Lloyd-Smith R, Turner A, Heath L, James N, et al. Bone mineral density, rib pain and other features of the female athlete triad in elite lightweight rowers. BMJ Open 2014;4(2):e004369. doi:[10.1136/bmjopen-2013-004369]

28.	de Borja C, Holtzman B, McCall LM, Carson TL, Moretti LJ, Farnsworth N, et al. Specific dietary practices in female athletes and their association with positive screening for disordered eating. Journal of Eating Disorders 2021;9(1):doi:[10.1186/s40337-021-00407-7]

29.	Amoruso I, Fonzo M, Barro A, Scardina C, Titton F, Bertoncello C, et al. Determinants of menstrual dysfunction in the female athlete triad: A cross-sectional study in Italian athletes. Psychology of Sport and Exercise 2024;73(doi:[10.1016/j.psychsport.2024.102653]

30.	Courtney A, Wyse A, Murphy B, McCarthy E, Kgosidialwa O, Tuthill A. Screening for risk of low energy availability in female Gaelic game athletes in Ireland. Irish Journal of Medical Science 2025;doi:[10.1007/s11845-025-04005-3]

31.	Meyer A, Haigis D, Klos B, Zipfel S, Resmark G, Rall K, et al. Relative Energy Deficiency in Sport—Multidisciplinary Treatment in Clinical Practice. Nutrients 2025;17(2):228. doi:[10.3390/nu17020228]

32.	Heikura IA, McCluskey WT, Tsai M-C, Johnson L, Murray H, Mountjoy M, et al. Application of the IOC Relative Energy Deficiency in Sport (REDs) Clinical Assessment Tool version 2 (CAT2) across 200+ elite athletes. British Journal of Sports Medicine 2025;59(1):24-35. doi:[10.1136/bjsports-2024-108121]
